# Sirt2 promotes white matter oligodendrogenesis during development and in models of neonatal hypoxia

**DOI:** 10.1038/s41467-022-32462-2

**Published:** 2022-08-15

**Authors:** Beata Jablonska, Katrina L. Adams, Panagiotis Kratimenos, Zhen Li, Emma Strickland, Tarik F. Haydar, Katharina Kusch, Klaus-Armin Nave, Vittorio Gallo

**Affiliations:** 1 Center for Neuroscience Research, Children’s National Research Institute, Children’s National Hospital, Washington, DC 20010 USA; 2 Neonatology Department, Children’s National Hospital, Washington, DC 20010 USA; 3 Max Planck Institute of Experimental Medicine, Department of Neurogenetics, Gottingen, Germany

**Keywords:** Oligodendrocyte, Glial progenitors

## Abstract

Delayed oligodendrocyte (OL) maturation caused by hypoxia (Hx)-induced neonatal brain injury results in hypomyelination and leads to neurological disabilities. Previously, we characterized Sirt1 as a crucial regulator of OL progenitor cell (OPC) proliferation in response to Hx. We now identify Sirt2 as a critical promoter of OL differentiation during both normal white matter development and in a mouse model of Hx. Importantly, we find that Hx reduces Sirt2 expression in mature OLs and that Sirt2 overexpression in OPCs restores mature OL populations. Reduced numbers of Sirt2^+^ OLs were also observed in the white matter of preterm human infants. We show that Sirt2 interacts with p27^Kip1^/FoxO1, p21^Cip1^/Cdk4, and Cdk5 pathways, and that these interactions are altered by Hx. Furthermore, Hx induces nuclear translocation of Sirt2 in OPCs where it binds several genomic targets. Overall, these results indicate that a balance of Sirt1 and Sirt2 activity is required for developmental oligodendrogenesis, and that these proteins represent potential targets for promoting repair following white matter injury.

## Abstract (web summary)

Neonatal hypoxia leads to white matter hypomyelination due to delayed oligodendrocyte maturation. The authors identify Sirt2 as a crucial regulator of oligodendrocyte differentiation during normal white matter development and in response to hypoxia.

## Introduction

Changes in myelination due to diffuse white matter injury (DWMI) are a common consequence of premature birth, periventricular leukomalacia, and congenital cardiac and respiratory disorders^1–4^. Hypoxia (Hx) damage during the neonatal period can lead to motor disabilities and cognitive deficits with long-term consequences, including cerebral palsy, intellectual disability, or epilepsy, which are often due to cellular and functional abnormalities^5,6^. Our laboratory has investigated cellular and molecular mechanisms of white matter (WM) alterations caused by developmental Hx. Common cellular hallmarks of Hx-induced cerebral WM injuries are increased proliferation of oligodendrocyte progenitor cells (OPCs), depletion of oligodendrocytes (OLs), and/or their delayed maturation, leading to improper myelination of callosal axons^7–10^. Myelin repair engages the generation of new mature OLs from OPCs, a process governed by intracellular factors whose expression and/or function may be impaired under Hx conditions. Understanding these molecular mechanisms will help to overcome therapeutic obstacles to successful repair following Hx injury.

Sirtuins are the main NAD-dependent deacetylases that utilize NAD^+^ in deacetylation reactions^11–13^. The deacetylase activity of the sirtuins is controlled by the cellular NAD^+^/NADH^+^ ratio, where NAD^+^ works as an activator, whereas NADH^+^ inhibits their activity. The seven mammalian homologs of sirtuins (Sirt1-7) play distinct roles in many physiological and pathological processes, due to their different subcellular localizations^14,15^. Previously, we found that nuclear Sirt1 is primarily expressed in OPCs in the subcortical WM, where it controls OPC proliferation during postnatal development, and promotes the regenerative response of OPCs after neonatal Hx, through regulation of the Cdk2 pathway^16^. Conversely, Sirt2, regulated by the QKI-dependent pathway, is expressed by mature OLs in the central nervous system and has been shown to localize to myelin sheaths^17–19^. Cytoplasmic Sirt2 deacetylates multiple proteins, including alpha-tubulin in cultured OPCs^19^ and HeLa cells^20^, as well as the polarity protein Par-3 in Schwann cells^21^. Sirt2 also suppressed PDGFRα expression after translocation to the nucleus, promoting differentiation of the CG4 glial cell line^22^. In addition, a recent study found that Sirt2 is delivered to axons from OL to promote mitochondrial ATP production^23^. Together, these studies suggest that Sirt2 plays multiple roles in OL physiology; however, the developmental role of Sirt2 in OL differentiation in vivo remains poorly understood. Furthermore, whether Sirt2 also plays a role in OL recovery following injury remains unknown, as does the relationship between Sirt1 and Sirt2 in regulating OL lineage dynamics.

To address these questions, we investigated the function of Sirt2 in subcortical WM development and recovery from chronic neonatal Hx. We find that Sirt2 is primarily expressed by mature OLs in the healthy WM and this expression is strongly downregulated following Hx. Using both loss-of-function and gain-of-function experiments in vivo, we identify Sirt2 as a critical promoter of OL differentiation during both normal WM development and after Hx. Interestingly, we also show that overexpression of Sirt2 in OPCs, but not mature OLs, restores OL populations after Hx through enhanced OPC proliferation and protection from apoptosis. Sirt2 interacts with multiple cell cycle proteins, including the p27^Kip1^/FoxO1 and p21^Cip1^/Cdk4 pathways, in normoxic (Nx) WM—and these interactions are altered following Hx. Importantly, we also demonstrate that Hx induces nuclear translocation of Sirt2 in OPCs, and we identify several genomic targets of Sirt2. Finally, we examine the relationship of Sirt1 and Sirt2 in OL development and recovery after Hx, and show that Sirt1 knockdown elevates Sirt2 expression and increases oligodendrogenesis.

## Results

### Developmental pattern of Sirt2 expression in normal and Hx WM

**Fig. 1:**
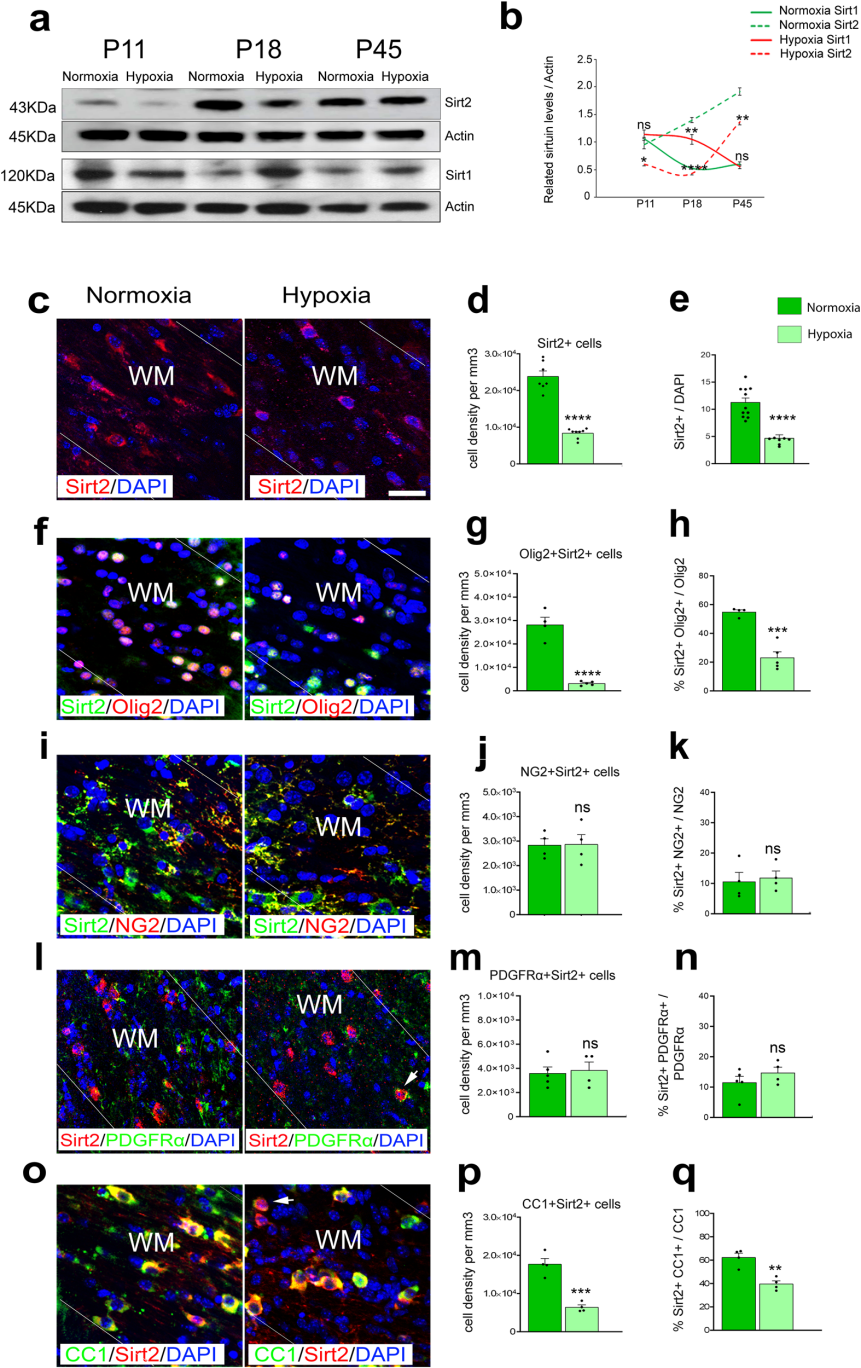
Hx reduces Sirt2 expression in mature WM OLs. **a** Representative western blots for Sirt2 and Sirt1 proteins in subcortical WM of Nx and Hx animals at postnatal days P11, P18, and P45. **b** Quantification of western blots. Graph displays mean ± SEM values (*n* = 3 brains per condition). At P11: Sirt1 ns *p* = 0.04793, Sirt2 *p = 0.0108; at P18: Sirt1 **p = 0.0044, Sirt2 ****p < 0.0001; at P45: Sirt1 ns *p* = 0.2347, Sirt2 **p = 0.0033 (Student’s *t* test). **c**, **f**, **i**, **l**, **o** Coronal sections of subcortical WM stained for Sirt2^+^ **c**, Sirt2^+^Olig2^+^ **f**, Sirt2^+^NG2^+^ **i**, Sirt2^+^PDGFRα^+^ **l**, and CC1^+^Sirt2^+^ **o** cells in Nx and Hx mice at P18. Dotted lines delineate WM. WM, white matter. Arrows point to nuclear Sirt2^+^ staining. Scale bar = 100 µm. **d**, **e** Quantification of the total Sirt2^+^ cell density **d** and percentage of Sirt2^+^ cells **e** in WM at P18 (****p < 0.0001 for **d**, **e**, *n* = 4 mice per group, Student’s *t* test). **g**, **h** Quantification of the total Olig2^+^Sirt2^+^ cell density (****p < 0.0001, n = 4 Nx and 5 Hx mice, Student’s *t* test) **g** and percentage of Sirt2^+^ OL lineage cells (***p = 0.0003, *n* = 4 Nx and 5 Hx mice, Student’s *t* test) **h** in WM at P18. **j**, **k** Quantification of the total NG2^+^Sirt2^+^ cell density (ns *p* = 0.9543, *n* = 4 per group, Student’s *t* test) **j** and percentage of Sirt2 expression in NG2^+^ OPCs in WM at P18 (ns *p* = 0.7479, *n* = 4 per group, Student’s *t* test) **k**. **m**, **n** Quantification of total PDGFRα^+^Sirt2^+^ cell density (ns *p* = 0.7748, *n* = 5 Nx and 4 Hx mice, Student’s *t* test) **m** and percentage of Sirt2 expression in PDGFRα^+^ OPCs (ns *p* = 0.9960, *n* = 5 Nx and 4 Hx mice, Student’s *t* test) **n**. **p**, **q** Quantification of the total CC1^+^Sirt2^+^ cell density (***p = 0.0004, *n* = 4 per group, Student’s *t* test) **p** and percentage of Sirt2^+^ mature CC1^+^-expressing OLs (**p = 0.0025, *n* = 4 per group, Student’s *t* test) **q** in WM at P18. Graphs display mean ± SEM values. All statistical tests are two-sided. Source data are provided as a Source Data file.

While sirtuins regulate diverse cellular processes^24^, their specific roles in OL development remain poorly understood. Previously, we found that Sirt1 is a crucial regulator of OPC proliferation^16^. On the other hand, Sirt2 has been shown to regulate cell differentiation^25–27^, suggesting that Sirt1 and Sirt2 may play sequential roles both during normal OL development and after injury. Western blot analysis demonstrated increasing levels of Sirt2 during postnatal development (P11, P18, and P45, (Nx, Fig. 1a, b). In contrast, Sirt1 expression decreased during this same period, indicating a reciprocal relationship between Sirt1 and Sirt2 expression in normal WM development. We next determined whether neonatal Hx modified this expression profile. Chronic neonatal Hx (postnatal day P3–P11) induces OL death and responsive OPC proliferation, resulting in a loss of mature CC1^+^ OLs and increase in NG2^+^ OPCs^7^. Hx altered both Sirt1 and Sirt2 protein levels at P11 and P18, with a return to normal expression by P45 (Fig. 1a, b). While Sirt1 expression was upregulated at P18 after Hx as previously demonstrated^16^, Sirt2 levels were downregulated both at P11 and P18. To confirm these changes in expression, we also quantified *Sirt1* and *Sirt2* transcript levels in FACS-purified OL lineage cells. *Sirt1* mRNA levels were significantly increased in purified NG2-expressing OPCs from Hx WM, compared to Nx controls (Supp. Fig. 1a). There was no significant change in *Sirt2* mRNA expression in NG2^+^ OPCs. Conversely, *Sirt2* mRNA expression was decreased in sorted CNP-EGFP^+^ OLs from Hx WM, with no change in *Sirt1* expression (Supp Fig. 1b). Therefore, Sirt1 is upregulated in OPCs, and Sirt2 is downregulated in OLs following neonatal Hx. As Hx causes a delay in OL maturation^7^, these results are consistent with a role of Sirt1 in OPC proliferation and/or suppression of differentiation^16^, and a potential role of Sirt2 in OL maturation during basal oligodendrogenesis and after WM damage.

We next analyzed Sirt2 protein expression within the OL lineage to determine whether neonatal Hx altered specific cell populations during postnatal WM development. Immunohistochemical labeling of coronal WM sections with anti-Sirt2 antibody demonstrated a reduction in the number of Sirt2^+^ cells after Hx insult (Fig. 1c–e). While the number of Sirt2^+^Olig2^+^ cells decreased after Hx (Fig. 1f, g), the NG2^+^Sirt2^+^ and PDGFRα^+^Sirt2^+^ cell populations remained unchanged (Fig. 1i, j, l, m). Furthermore, the percentage of Sirt2^+^Olig2^+^ cells within the Olig2^+^ cell population was significantly reduced after Hx, compared with Nx (Fig. 1h), but we did not observe any change in the percentage of Sirt2 cells within the NG2^+^ and PDGFRα^+^ populations, respectively (Fig. 1k, n). These results indicate that Hx may specifically affect the mature OL Sirt2^+^ population. In support of this notion, we found a significant reduction in the number of Sirt2^+^CC1^+^ mature OLs in WM after Hx, along with a decreased percentage of Sirt2^+^CC1^+^ cells within the CC1^+^ population (Fig. 1o–q). Finally, we did not observe any changes in the density or percentage of astrocytes and microglia expressing Sirt2 between Nx and Hx WM (Supp. Fig. 2a–f). Overall, these observations indicate that Sirt2 expression in OLs is associated with lineage progression, and that Hx reduces Sirt2 expression specifically in maturing OLs.

### Sirt2 expression is reduced in preterm infants

**Fig. 2:**
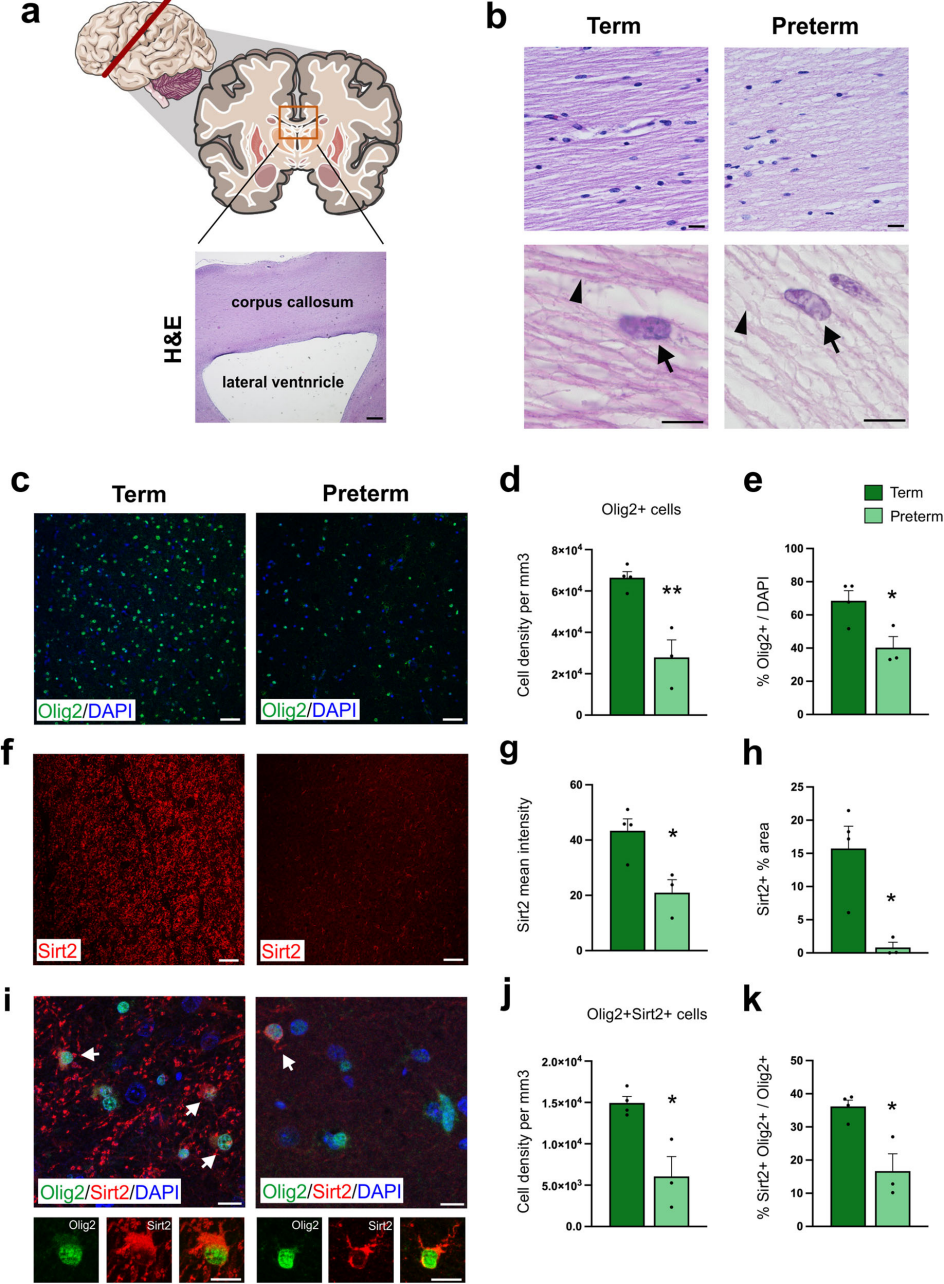
Reduced Sirt2 expression in subcortical WM of preterm infants. **a** Tissue sections from the corpus callosum of preterm human neonates and term controls were analyzed. H&E image shows lower magnification of corpus callosum region analyzed for preterm and term controls. Scale bar = 100 µm. **b** Representative H&E photomicrographs of corpus callosum (*n* = 4 term and 4 preterm). In term controls, well-defined OLs with well-defined nucleolus (arrow) and dense neuropil were observed (arrowhead). In contrast, in preterm neonates (right panels) hypodense and rarefied neuropil were present (arrowhead) with OLs that appear edematous and vacuolated (arrow). Scale bars = 50 µm for upper panels and 20 µm for lower panels. **c** Olig2^+^ immunostaining (green) in WM of term and preterm neonates. Scale bars = 50 µm. **d**, **e** Quantification of the density **d** and the percentage of Olig2^+^ cells **e** in term and preterm neonates. (**p = 0.005, *p = 0.026, *n* = 4 term and 3 preterm, Student’s *t* test). **f** Low magnification images of Sirt2^+^ immunostaining (red) in WM of term and preterm neonates. Scale bars = 50 µm. **g** Quantification of the intensity of Sirt2 staining in term and preterm neonates (*p = 0.017, *n* = 4 term and 3 preterm, Student’s *t* test). **h** Quantification of the percent area of Sirt2^+^ signal in the WM of term and preterm neonates (*p = 0.014, *n* = 4 term and 3 preterm, Student’s *t* test). **i** Sirt2 expression within WM Olig2^+^ cells of term and preterm neonates. Bottom panels show magnified single-channel images for Olig2 (green) and Sirt2 (red) to highlight cytoplasmic localization of Sirt2. Scale bars = 10 µm. **j** Quantification of the density of Sirt2^+^Olig2^+^ cells in term and preterm neonates (*p = 0.01, *n* = 4 term and 3 preterm, Student’s *t* test). **k** Quantification of the percentage of Sirt2^+^ oligodendrocytes in term and preterm neonates (*p = 0.011, *n* = 4 term and 3 preterm, Student’s *t* test). H&E, Hematoxylin and Eosin. Data are represented as mean ± SEM. All statistical tests are two-sided. Source data are provided as a Source Data file. The human brain schematic in **a** was created using Servier Medical Art templates, which are licensed under a Creative Commons Attribution 3.0 Unported License.

To determine whether Sirt2 is also expressed by developing human OLs, we analyzed brain tissue samples from human infants born at term (>37 weeks of gestation) and human preterm infants (<32 weeks of gestation) (Supp. Table 1). We first performed H&E histology analysis of the tissue samples, focusing on the corpus callosum and subcortical WM (Fig. 2a). In term controls, we observed dense neuropil and OLs with well-defined nucleolus, while preterm neonates presented a hypodense and rarefied neuropil with edematous and vacuolated OLs (Fig. 2b). We also observed a reduction in Olig2^+^ OL lineage cells in preterm neonates, compared to term controls (Fig. 2c–e). Sirt2 expression was dramatically reduced in preterm neonates, compared to term controls, both in the intensity and amount of staining (Fig. 2f–h). Lastly, we found a significant reduction in the number of Sirt2^+^Olig2^+^ cells within the WM of human preterm infants, compared to term controls (Fig. 2i–k). Together, these results indicate that Sirt2 is normally expressed by human OLs during perinatal brain development, and that premature birth significantly reduces Sirt2 expression in the WM.

### Sirt2 knockdown reduces oligodendrogenesis in vitro

To test whether Sirt2 expression is required for OL differentiation, both during normal development and after Hx, we performed siRNA-mediated knockdown of Sirt2 in Nx and Hx WM cultured cells (Supp. Fig. 3). Sirt2 siRNA strongly reduced Sirt2 protein levels in both Nx and Hx cultures (Supp. Fig. 3c). In scrambled siRNA controls, Hx itself caused a reduction in the percentage of Olig2^+^ and GalC^+^ cells (Supp. Fig. 3a, b, d, e), confirming a negative effect of Hx on OL differentiation in culture. Under Nx conditions, Sirt2 knockdown significantly reduced the percentages of Olig2^+^ and GalC^+^ cells, as compared to scrambled controls, and this effect was even more pronounced under Hx conditions (Supp. Fig. 3a, b, d, e). Altogether, these findings indicate that Sirt2 loss-of-function reduces oligodendrogenesis and that Hx-induced loss of Sirt2 expression may explain the delay in OL maturation commonly seen after neonatal injury.

### Modulation of Sirt2 levels in OL lineage in vivo

To investigate whether manipulating Sirt2 expression in vivo also regulates OL differentiation under Nx and Hx conditions, we performed loss- and gain-of-function experiments by crossing inducible CreERT mouse strains with either a Sirt2-floxed mouse strain or a Sirt2-overexpressing mouse strain (Supp. Fig. 4). In Sirt2^fl/fl^PDGFRα^CreERT^ and Sirt2^STOP^PDGFRα^CreERT^ mice, Sirt2 was ablated or overexpressed in PDGFRα-expressing OPCs, respectively, after two tamoxifen injections at P11 and P12. In Sirt2^STOP^PLP^CreERT^ mice, increased expression of Sirt2 occurred in PLP^+^ mature OLs following the same tamoxifen protocol. All three groups underwent the Hx paradigm (10.5% oxygen), along with wild-type (WT) controls (WT-PDGFRα^CreERT^ or WT-PLP^CreERT^), and cell quantification was performed at P18.

First, we verified changes in Sirt2 expression following tamoxifen-induced recombination by quantifying the total number of Sirt2^+^ cells in P18 WM, under Nx and Hx conditions. Under physiological conditions, the density of Sirt2^+^ cells was significantly reduced by Sirt2 ablation as compared to controls (Supp. Fig. 4a–c). Conversely, Sirt2 overexpression in both PDGFRα^+^ cells or PLP^+^ cells significantly increased the density of Sirt2^+^ cells in WM, compared to controls (Supp. Fig. 4a, d, e). Hx reduced the density of Sirt2^+^ WM cells in all mouse strains, as compared to Nx (Supp. Fig. 4a–e). However, the forced overexpression of Sirt2 in Sirt2^STOP^PDGFRα^CreERT^ and Sirt2^STOP^PLP^CreERT^ mice following Hx was sufficient to restore the density of Sirt2^+^ cells to WT Nx levels (Supp. Fig. 4d, e). Therefore, the percent reduction of Sirt2 expression after Hx varied across the different genotypes (Supp. Fig. 4f, g). To examine the levels of Sirt2 expression more closely within the OL lineage, we also quantified the number of Sirt2^+^PDGFRα^+^ OPCs within the WM across the different mouse strains, under both Nx and Hx conditions. As expected, the number of Sirt2^+^ OPCs decreased in the Sirt2^fl/fl^PDGFRα^CreERT^ mice, but increased in the Sirt2^STOP^PDGFRα^CreERT^ mice as compared to their Nx WT littermates (Supp. Fig. 4h, i). Sirt2^STOP^PLP^CreERT^ mice did not have significantly different numbers of Sirt2^+^ OPCs compared to WT-PLP^CreERT^ controls (Supp. Fig. 4j), confirming that Sirt2 overexpression was restricted to mature OLs in this strain.

Next, we asked whether Sirt2 knockdown also reduced oligodendrogenesis in vivo. WT-PDGFRα^CreERT^ and Sirt2^fl/fl^PDGFRα^CreERT^ mice were analyzed at P18 following our standard experimental protocol (Supp. Fig. 5a, b). Sirt2 ablation significantly reduced the number of Olig2^+^ OL lineage cells and CC1^+^ mature OLs in Nx WM and Hx further exacerbated these reductions (Supp. Fig. 5c–h). Therefore, Sirt2 ablation in PDGFRα-expressing OPCs does reduce the production of OLs in vivo, both under Nx and Hx conditions.

### Sirt2 overexpression selectively protects PDGFRα-expressing OPCs against Hx-induced cell death

**Fig. 3:**
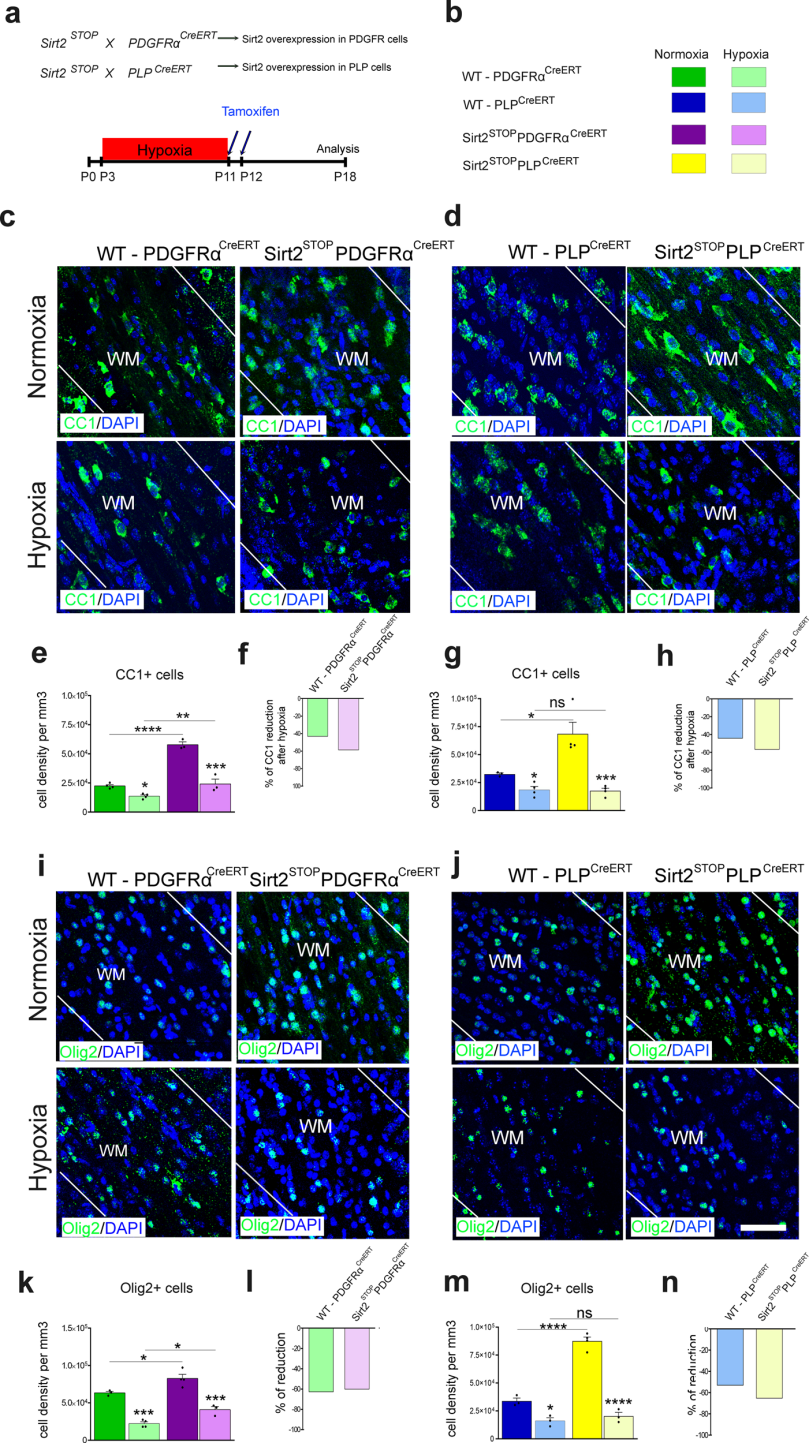
Sirt2 overexpression alters oligodendrogenesis in vivo. **a**, **b** Experimental approach for genetic Sirt2 overexpression in PDGFRα^+^ OPCs or PLP^+^ mature OLs in combination with Hx paradigm. **c**, **d** Coronal sections of subcortical WM stained for CC1 from Sirt2^STOP^PDGFRα^CreERT^ **c** and Sirt2^STOP^PLP^CreERT^ **d** transgenic mice, with respective controls, after Nx and Hx. White lines delineate WM. WM, white matter. **e**, **g** Quantification of the total CC1^+^ cell density in WM at P18 (WT: Nx vs Hx *p = 0.0433, Sirt2^STOP^PDGFRα^CreERT^: Nx vs Hx ***p = 0.0005, WT Nx vs Sirt2^STOP^PDGFRα^CreERT^ Nx ****p < 0.0001, WT Hx vs Sirt2^STOP^PDGFRα^CreERT^ Hx **p = 0.0031, *n* = 4 WT-Nx, 3 WT-Hx, 3 Sirt2^STOP^-Nx, 3 Sirt2^STOP^-Hx mice, ANOVA with Tukey’s multiple comparisons adjustment) **e**, and (WT: Nx vs Hx *p = 0.0491, Sirt2^STOP^PLP^CreERT^: Nx vs Hx ***p = 0.0002, WT Nx vs Sirt2^STOP^PLP^CreERT^ Nx *p = 0.0176, WT Hx vs Sirt2^STOP^PLP^CreERT^ Hx ns = 0.8791, *n* = 3 WT-Nx, 4 WT-Hx, 4 Sirt2^STOP^-Nx, 4 Sirt2^STOP^-Hx mice, ANOVA with Tukey’s multiple comparisons adjustment) **g**. **i**, **j** Coronal sections of subcortical WM stained for Olig2 from Sirt2^STOP^PDGFRα^CreERT^ **i** and Sirt2^STOP^PLP^CreERT^ **j** transgenic mice, with respective controls, after Nx and Hx. Scale bar = 100 µm. **k**, **m** Quantification of the total Olig2^+^ cell density in WM at P18 (WT: Nx vs Hx ***p = 0.0002, Sirt2^STOP^PDGFRα^CreERT^: Nx vs Hx ***p = 0.0001, WT Nx vs Sirt2^STOP^PDGFRα^CreERT^ Nx *p = 0.0263, WT Hx vs Sirt2^STOP^PDGFRα^CreERT^ Hx *p = 0.0357, *n* = 3 WT-Nx, 4 WT-Hx, 4 Sirt2^STOP^-Nx, 3 Sirt2^STOP^-Hx mice, ANOVA with Tukey’s multiple comparisons adjustment) **k**, and (WT: Nx vs Hx *p = 0.0219, Sirt2^STOP^PLP^CreERT^: Nx vs Hx ****p < 0.0001, WT Nx vs Sirt2^STOP^PLP^CreERT^ Nx ****p < 0.0001, WT Hx vs Sirt2^STOP^PLP^CreERT^ Hx ns = 0.8177, *n* = 3 WT-Nx, 3 WT-Hx, 4 Sirt2^STOP^-Nx, 3 Sirt2^STOP^-Hx mice, ANOVA with Tukey’s multiple comparisons adjustment) **m**. **f**, **h**, **l**, **n** Quantification of the percent of reduction of CC1^+^ and Olig2^+^ cells after Hx in each transgenic mouse strain. No changes were found in percent of CC1^+^ and Olig2^+^ cell reduction in WM of Sirt2^STOP^PDGFRα^CreERT^ or Sirt2^STOP^PLP^CreERT^ and their WT littermates. All graphs display mean ± SEM values, except for percent reduction. All statistical tests are two-sided. Source data are provided as a Source Data file.

We next analyzed the effects of Sirt2 overexpression in PDGFRα-expressing OPCs or mature PLP-expressing OLs under Nx and Hx conditions (Fig. 3a, b). Both Sirt2^STOP^PDGFRα^CreERT^ and Sirt2^STOP^PLP^CreERT^ mice had significantly higher numbers of CC1^+^ cells and Olig2^+^ cells under Nx conditions, compared to WT controls (Fig. 3c–e, g, i–k, m). Again, this indicates that Sirt2 promotes oligodendrogenesis during normal WM development. Since Hx decreased Sirt2^+^ cells in the WM of WT animals (Fig. 1d, e), we next determined whether Sirt2 overexpression in PDGFRα-expressing and PLP-expressing cells was sufficient to restore Sirt2 cell number. The number of Sirt2^+^ cells in both Sirt2^STOP^PDGFRα^CreERT^ and Sirt2^STOP^PLP^CreERT^ mice after Hx was significantly higher than in Hx-treated WT mice and was comparable to Nx WT mice (Supp. Fig. 4d, e), indicating rescue of the Sirt2^+^ cell population. However, the effects of Sirt2 overexpression on the OL lineage following Hx varied greatly between the different mouse strains. First, Sirt2 overexpression in either PDGFRα- or PLP-expressing cells did not prevent a Hx-induced decrease of total CC1^+^ or Olig2^+^ OLs, compared to Nx Sirt2^STOP^ mice (Fig. 3c–e, g, i–k, m). This was confirmed by analyzing the percentage of reduction after Hx in each mouse strain (Fig. 3f, h, l, n). However, Sirt2 overexpression in OPCs following Hx did significantly increase the number of CC1^+^ and Olig2^+^ cells, compared to WT Hx mice (Fig. 3c, e, i, k).

To further analyze the effect of Sirt2 overexpression on OL differentiation under Nx and Hx conditions, we then quantified the percentage and total number of Olig2^+^ OL lineage cells expressing Enpp6. Enpp6 is highly expressed by newly-generated OLs and expressed at low levels in mature CC1^+^ OLs^28^ making it a useful marker to distinguish between the two cell populations (Supp. Fig. 6a). We found that Hx reduced the percentage and total number of both *Enpp6*^+^-high and *Enpp6*^+^-low OLs compared to Nx controls (Supp. Fig. 6b–g), as expected from our previous analysis. Interestingly, Sirt2 overexpression in OPCs following Nx significantly increased the percentage and total number of *Enpp6*^+^-low mature OLs, but did not increase the percentage nor total number of *Enpp6*^+^-high newly-generated OLs, as compared to WT Nx controls (Supp. Fig. 6d–g). This suggests that Sirt2 overexpression in OPCs under Nx conditions primarily promotes OL maturation. Importantly, however, we found that Sirt2 overexpression in OPCs following Hx rescues both *Enpp6*^+^-high and *Enpp6*^+^-low OL populations, as compared to WT Hx mice (Supp. Fig. 6d–g).

This suggests that overexpression of Sirt2 in OPCs increases oligodendrogenesis after Hx, possibly by protecting Sirt2-expressing OPCs from Hx-induced cell death. Indeed, analysis of apoptosis identified a significantly reduced total number of Caspase3^+^ cells and Caspase3^+^PDGFRα^+^ OPCs in Sirt2^STOP^PDGFRα^CreERT^ mice after Hx, compared to WT Hx controls (Supp. Fig. 7b, f, g). Sirt2 overexpression in OPCs also decreased apoptosis of mature CC1^+^ OLs (Supp. Fig. 7c, h, i). Finally, to determine whether Sirt2 overexpression in PDGFRα-expressing OPCs mimicked Sirt1’s pro-proliferative role, we analyzed cell proliferation by administering BrdU to mice 2 h prior to sacrifice to label dividing cells in the WM. As expected, OPC proliferation increased in WT mice following Hx (Supp. Fig. 7a, d, e)^7^. Sirt2 overexpression increased the number of proliferating OPCs in both Nx and Hx conditions (Supp. Fig. 7a, d, e, j). Therefore, Sirt2 overexpression in OPCs restores OL numbers after Hx through both increased proliferation and protection from cell death.

In contrast to Sirt2^STOP^PDGFRα^CreERT^ mice, Sirt2 overexpression in PLP^+^ mature OLs did not rescue the Hx-induced loss of CC1^+^ and Olig2^+^ cells in WM (Fig. 3d, g, j, m). Interestingly, this occurred despite a large increase in oligodendrogenesis in Sirt2^STOP^PLP^CreERT^ mice under Nx conditions (Fig. 3d, g, h, j, m, n). This suggests that targeting Sirt2 expression in mature OLs occurs too late during lineage maturation to exert a protective effect against Hx-induced cell death. Indeed, we did not see any differences in total apoptotic cells in Sirt2^STOP^PLP^CreERT^ mice, compared to WT mice (Nx: Sirt2^STOP^PLP^CreERT^; 13.3 ± 6.05 × 10^3^cells/mm^3^ vs. WT-PLP^CreERT^; 14.4 ± 10.44 × 10^3^cells/mm^3^, Hx: Sirt2^STOP^PLP^CreERT^; 18.9 ± 9.69 × 10^3^cells/mm^3^ vs. WT-PLP^CreERT^; 19.1 ± 3.06 × 10^3^ cells/mm^3^). Altogether, these data suggest that Sirt2 overexpression in OPCs prevents Hx-induced cell death, whereas Sirt2 overexpression in more mature PLP^+^-expressing OLs does not protect OL lineage cells from Hx insult.

### Sirt2 overexpression in PDGFRα^+^ OPCs, but not PLP^+^ cells, maintains Sirt2^+^ OLs against Hx injury

**Fig. 4:**
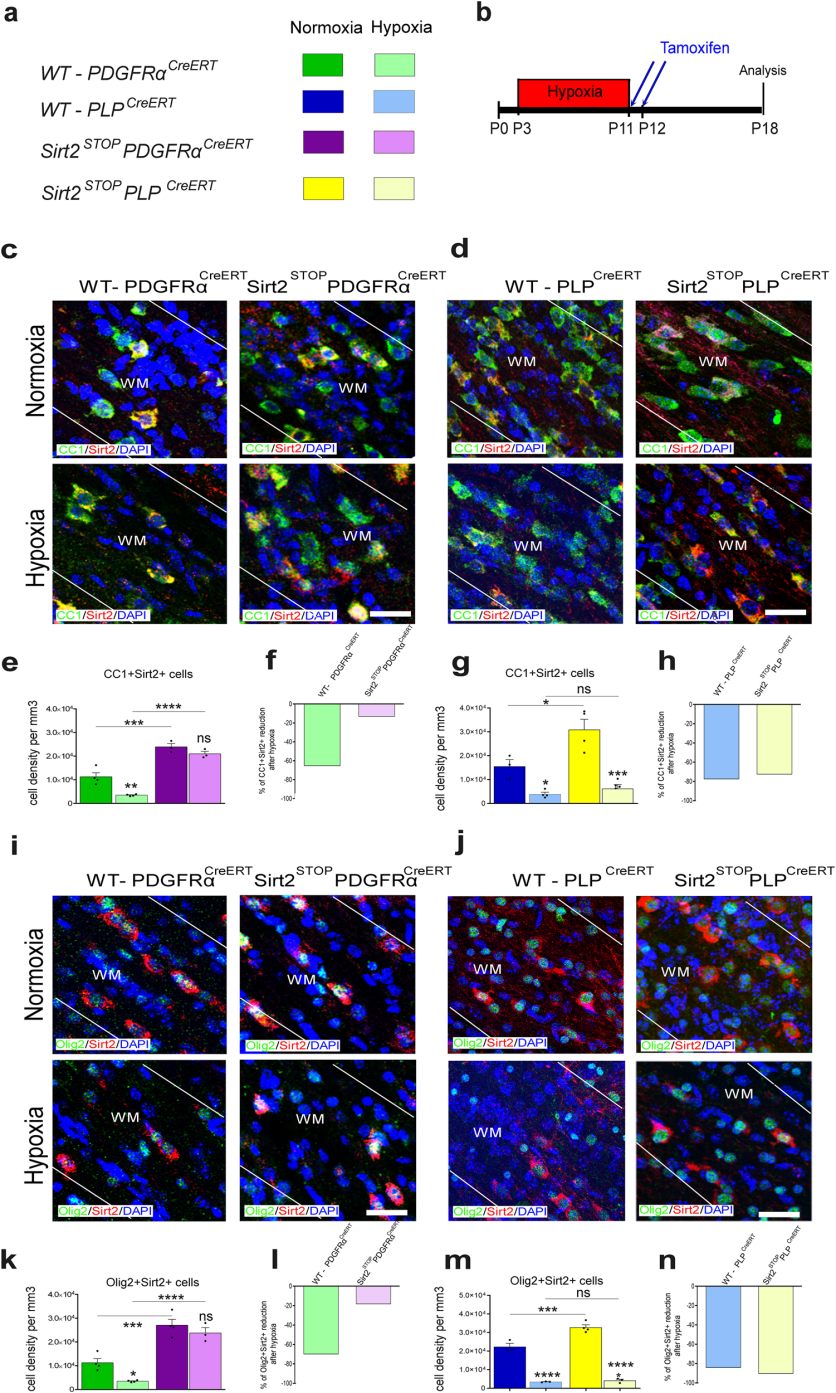
Sirt2^+^ OLs are protected from Hx only in Sirt2^STOP^PDGFRα^CreERT^ mice. **a** Color legend for different transgenic mice in Nx and Hx. **b** Experimental Hx paradigm. **c**, **d** Coronal sections of subcortical WM stained for CC1 and Sirt2 from Sirt2^STOP^PDGFRα^CreERT^ **c** and Sirt2^STOP^PLP^CreERT^ **d** transgenic mice, with respective controls, after Nx and Hx. White lines delineate WM, WM-white matter. Scale bar = 100 µm. **e**, **g** Quantification of the total CC1^+^Sirt2^+^ cell density in WM at P18 (WT: Nx vs Hx **p = 0.0037, Sirt2^STOP^PDGFRα^CreERT^: Nx vs Hx ns = 0.3774, WT Nx vs Sirt2^STOP^PDGFRα^CreERT^ Nx ***p = 0.0002, WT Hx vs Sirt2^STOP^PDGFRα^CreERT^ Hx ****p < 0.0001, *n* = 4 WT-Nx, 4 WT-Hx, 3 Sirt2^STOP^-Nx, 3 Sirt2^STOP^-Hx mice, ANOVA with Tukey’s multiple comparisons adjustment) **e**, and (WT: Nx vs Hx *p = 0.0220, Sirt2^STOP^PLP^CreERT^: Nx vs Hx ***p = 0.0002, WT Nx vs Sirt2^STOP^PLP^CreERT^ Nx *p = 0.0104, WT Hx vs Sirt2^STOP^PLP^CreERT^ Hx ns = 0.5991, *n* = 3 WT-Nx, 4 WT-Hx, 4 Sirt2^STOP^-Nx, 4 Sirt2^STOP^-Hx mice, ANOVA with Tukey’s multiple comparisons adjustment) **g**. **i**, **j** Coronal sections of subcortical WM stained for Olig2 and Sirt2 from Sirt2^STOP^PDGFRα^CreERT^ **i** and Sirt2^STOP^PLP^CreERT^ **j** transgenic mice, with respective controls, after Nx and Hx. Scale bar = 100 µm. **k**, **m** Quantification of the total Olig2^+^Sirt2^+^ cell density in WM at P18 (WT: Nx vs Hx *p = =0.0415, Sirt2^STOP^PDGFRα^CreERT^: Nx vs Hx ns = 0.3589, WT Nx vs Sirt2^STOP^PDGFRα^CreERT^ Nx ***p = 0.0001, WT Hx vs Sirt2^STOP^PDGFRα^CreERT^ Hx ****p < 0.0001, *n* = 3 WT-Nx, 4 WT-Hx, 4 Sirt2^STOP^-Nx, 3 Sirt2^STOP^-Hx mice, ANOVA with Tukey’s multiple comparisons adjustment) **k**, and (WT: Nx vs Hx ****p < 0.001, Sirt2^STOP^PLP^CreERT^: Nx vs Hx ****p = 0.0001, WT Nx vs Sirt2^STOP^PLP^CreERT^ Nx ***p = 0.0009 WT Hx vs Sirt2^STOP^PLP^CreER^ Hx ns = 0.9780, *n* = 3 WT-Nx, 3 WT-Hx, 4 Sirt2^STOP^-Nx, 3 Sirt2^STOP^-Hx mice, ANOVA with Tukey’s multiple comparisons adjustment) **m**. **f**, **h**, **l**, **n** Quantification of the percent of reduction of CC1^+^Sirt2^+^ and Olig2^+^Sirt2^+^ cells after Hx in each transgenic mouse strain. Changes in the percentage of CC1^+^Sirt2^+^ and Olig2^+^Sirt2^+^ cell reduction after Hx were found only in WM of Sirt2^STOP^PDGFRα^CreERT^, but not Sirt2^STOP^PLP^CreERT^ mice. All graphs display mean ± SEM values, except for percent reduction. All statistical tests are two-sided. Source data are provided as a Source Data file.

To investigate a protective role of Sirt2 expression following Hx, we next defined changes specifically in Sirt2^+^ OLs in two transgenic mouse strains, Sirt2^STOP^PDGFRα^CreERT^ and Sirt2^STOP^PLP^CreERT^ mice. Under Nx conditions, Sirt2 overexpression in OPCs considerably increased the density of CC1^+^Sirt2^+^ and Olig2^+^Sirt2^+^ cells in WM, as compared to Nx WT-PDGFRα controls (Fig. 4a–c, e, i, k). Furthermore, the density of Sirt2^+^ OLs was not significantly altered in Sirt2^STOP^PDGFRα^CreERT^ mice following Hx, and was comparable to Nx WT levels (Fig. 4e, k). Thus, in comparing percent reduction, Hx had a drastically reduced effect on CC1^+^Sirt2^+^ and Olig2^+^Sirt2^+^ cells when Sirt2 was overexpressed in OPCs (Fig. 4f, l).

Interestingly, we previously found a decrease in total CC1^+^ cells in Sirt2^STOP^PDGFRα^CreERT^ mice after Hx (Fig. 3e). This discrepancy suggests that not all CC1^+^ OLs express Sirt2. To investigate the distribution of Sirt2 in the CC1^+^ population, we quantified CC1^+^ cells that were negative for Sirt2. Interestingly, approximately 50% of CC1^+^ cells expressed Sirt2 in WT mice under Nx conditions (Supp. Fig. 8a, b). Following Hx, this decreased to ∼30%, in agreement with our finding that Hx induces a decrease in Sirt2 expression. Overall, in Sirt2^STOP^PDGFRα^CreERT^ mice there was a larger proportion (∼70%) of CC1^+^Sirt2^+^ cells, and consequently, a smaller percentage of CC1^+^ OLs that did not express Sirt2 under Nx conditions (Supp. Fig. 8a, b). Interestingly, in Sirt2^STOP^PDGFRα^CreERT^ mice, the percentage of CC1^+^ cells that did not express Sirt2 was further reduced after Hx (Supp. Fig. 8a, b). These findings suggest that overexpression of Sirt2 at an early developmental stage (PDGFRα^+^ OPCs) prevents the loss of Sirt2^+^CC1^+^ mature OLs, which are less susceptible to Hx damage than Sirt2 negative OLs.

Similar to Sirt2 overexpression in PDGFRα^+^ cells, Sirt2 overexpression in PLP^+^ cells increased the number of CC1^+^Sirt2^+^ and Olig2^+^Sirt2^+^ cells compared to WT controls under Nx conditions (Fig. 4d, g, j, m). In contrast to Sirt2^STOP^PDGFRα^CreERT^ mice, Hx still induced a considerable reduction in CC1^+^Sirt2^+^ and Olig2^+^Sirt2^+^ cells in Sirt2^STOP^PLP^CreERT^ mice (Fig. 4d, g, j, m). Therefore, the percentage of reduction in CC1^+^Sirt2^+^ and Olig2^+^Sirt2^+^ cells induced by Hx was similar in Sirt2^STOP^PLP^CreERT^ and WT mice (Fig. 4h, n), indicating that Sirt2 overexpression in PLP^+^ cells did not protect OLs from Hx insult. Together, these results point to a crucial role of Sirt2 in OL differentiation during the developmental transition from proliferating progenitor to post-mitotic OLs.

### Sirt2 interacts with multiple cell cycle proteins

**Fig. 5:**
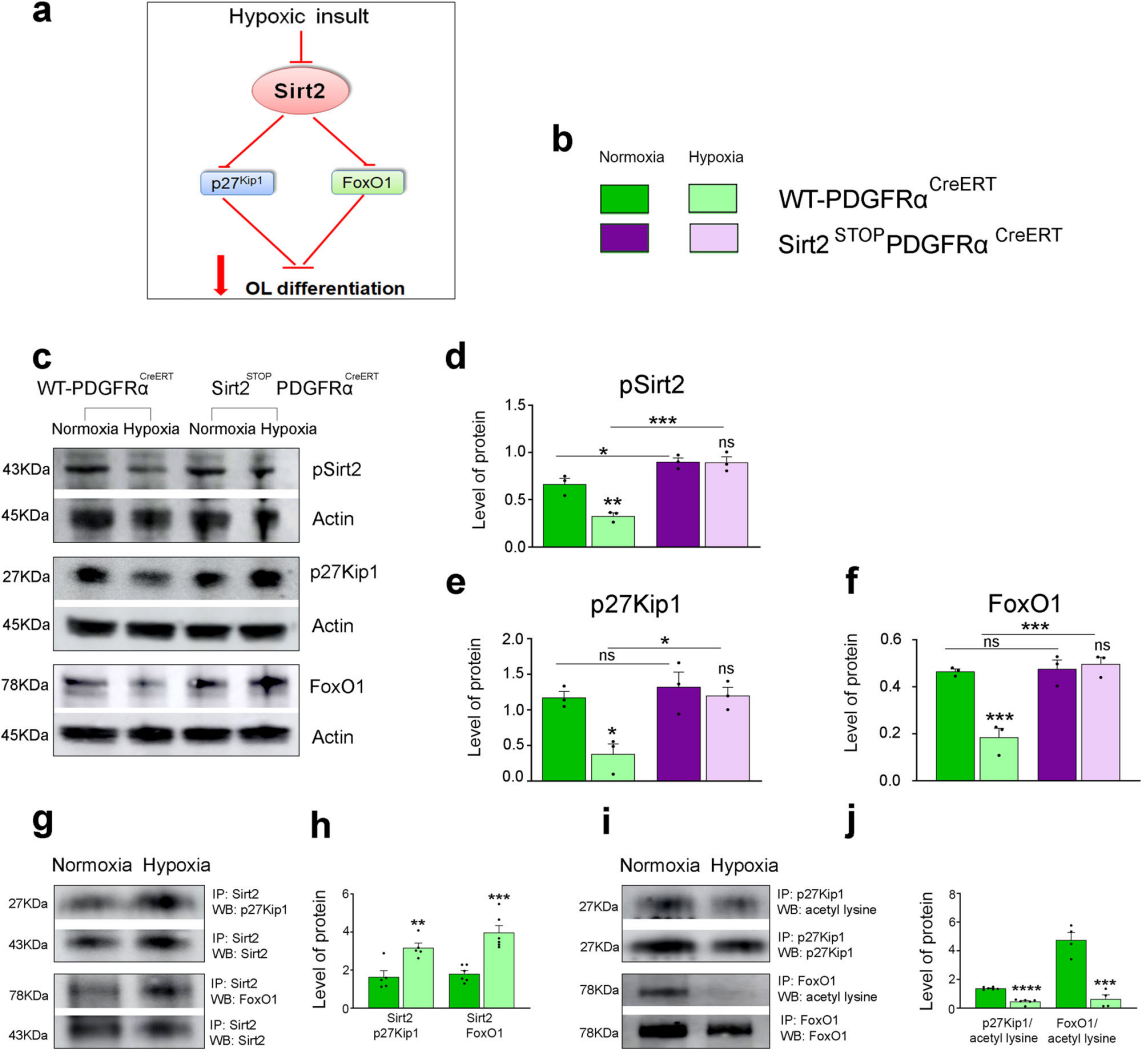
Hx increases the interaction of Sirt2 with FoxO1 and p27^Kip1^ in WM. **a** Cartoon depicting the FoxO1/p27^Kip1^ pathway regulated by Sirt2. **b** Color legend for different transgenic mice in Nx and Hx. **c** Western blots for expression of phosphorylated Sirt2 at Ser331, p27^Kip1^, and FoxO1 proteins in WM lysates of Sirt2^STOP^PDGFRα^CreERT^ mice and their WT littermates. **d**–**f** Quantification of protein levels of pSirt2 **d**, p27^Kip1^ **e**, and FoxO1 **f** in Nx and Hx WM of WT and Sirt2^STOP^PDGFRα^CreERT^ mice (pSirt2: **p = 0.0064, *p = 0.0428, ***p = 0.0002; p27^Kip1^: Nx vs Hx *p = 0.0200, Hx vs Hx *p = 0.0168; FoxO1: Nx vs Hx ***p = 0.0009, Hx vs Hx ***p = 0.0004; *n* = 3 per group, ANOVA with Tukey’s multiple comparisons adjustment). Graphs display mean ± SEM values. **g** Co-immunoprecipitation of dissected WM with Sirt2 antibody followed by Western blot for p27^Kip1^ and FoxO1, respectively to detect Sirt2 protein interactions. Protein complexes were identified by their sizes (27KD and 78-82KD, respectively). **i** Western blots for acetyl lysine levels of p27^Kip1^ and FoxO1 proteins. **h**, **j** Quantification of co-immunoprecipitation results for Sirt2/p27^Kip1^ (**p = 0.0054), Sirt2/FoxO1 (***p = 0.0003) **h**, acetyl lysine p27^Kip1^ (****p < 0.0001), acetyl lysine FoxO1 (***p = 0.0005), **j** (*n* = 5 Nx and Hx brains for Sirt2/p27, 6 Nx and Hx brains for Sirt2/Foxo1, 6 Nx and Hx brains for p27 acetyl, 4 Nx and Hx brains for Foxo1 acetyl, all Student’s *t* tests). Graphs display mean ± SEM values. All statistical tests are two-sided. Source data are provided as a Source Data file.

We next investigated potential molecular mechanisms through which Sirt2 regulates OL differentiation, first focusing on the interactions of cytoplasmic Sirt2 in mature OLs (Fig. 5a, b). Since previous studies have demonstrated that Sirt2 phosphorylation at Ser331 alters its enzymatic activity^29^, we first determined whether Hx altered Sirt2 phosphorylation. We found reduced levels of phosphorylated Sirt2 in the WM of WT mice after Hx, as compared to Nx lysates (Fig. 5c, d). Interestingly, Sirt2 overexpression in OPCs increased the levels of phosphorylated Sirt2 in both Nx and Hx WM, as compared to WT controls (Fig. 5c, d). These results suggest that Sirt2’s protein interactions and enzymatic activity may change following Hx.

The cyclin inhibitors, p27^Kip1^ and p21^Cip1^ govern two distinct signaling pathways (FoxO1/p27^Kip1^ and Cdk4/p21^Cip1^) and are involved in OL differentiation^30–33^. Previously, we demonstrated that OL regeneration after Hx is mediated by the FoxO1/p27^Kip1^ pathway^7^. Neonatal Hx caused a significant reduction in the expression of total p27^Kip1^, FoxO1, and FoxO3a proteins^7^. Additionally, gain-of-function experiments, in which FoxO1 or p27^Kip1^ were overexpressed in Hx WM, showed significantly higher percentages of OLs expressing O4, GalC, and Olig2 in cultures, as well as CC1, S100β, and MBP in vivo^7^. Therefore, we investigated whether Sirt2 regulated these G1/S and G2/M checkpoints in OPCs, promoting cell cycle exit during normal WM development and following neonatal Hx.

We first assessed the protein levels of p27^Kip1^ and FoxO1 in the WM of WT and Sirt2^STOP^PDGFRα^CreERT^ mice after Nx and Hx. We confirmed that both p27^Kip1^ and FoxO1 were reduced in WT mice after Hx, and interestingly, these reductions were rescued by Sirt2 overexpression in OPCs (Fig. 5c, e, f). To assess whether Sirt2 directly interacts with p27^Kip1^ and FoxO1, we then performed co-immunoprecipitation. Under Nx conditions, a Sirt2/p27^Kip1^ complex formed, and expression of this complex was elevated after Hx (Fig. 5g, h). This also corresponded to reduced p27^Kip1^ acetylation after Hx (Fig. 5i, j), suggesting increased deacetylation of p27^Kip1^, which likely leads to reduced stability and increased degradation of p27^Kip1^. We also observed the presence of a Sirt2/FoxO1 complex under Nx conditions, which was elevated after Hx (Fig. 5g, h). This also corresponded with increased deacetylation of FoxO1 (Fig. 5i, j), again indicating increased degradation of FoxO1. Together, these results demonstrate that both Hx and Sirt2 overexpression impact the level of Sirt2 phosphorylation, p27^Kip1^, and FoxO1. These findings also suggest that Sirt2 regulates the stability of both p27^Kip1^ and FoxO1, and that Hx alters these interactions, resulting in lower levels of p27^Kip1^ and FoxO1, leading to a reduction in OL differentiation.

**Fig. 6:**
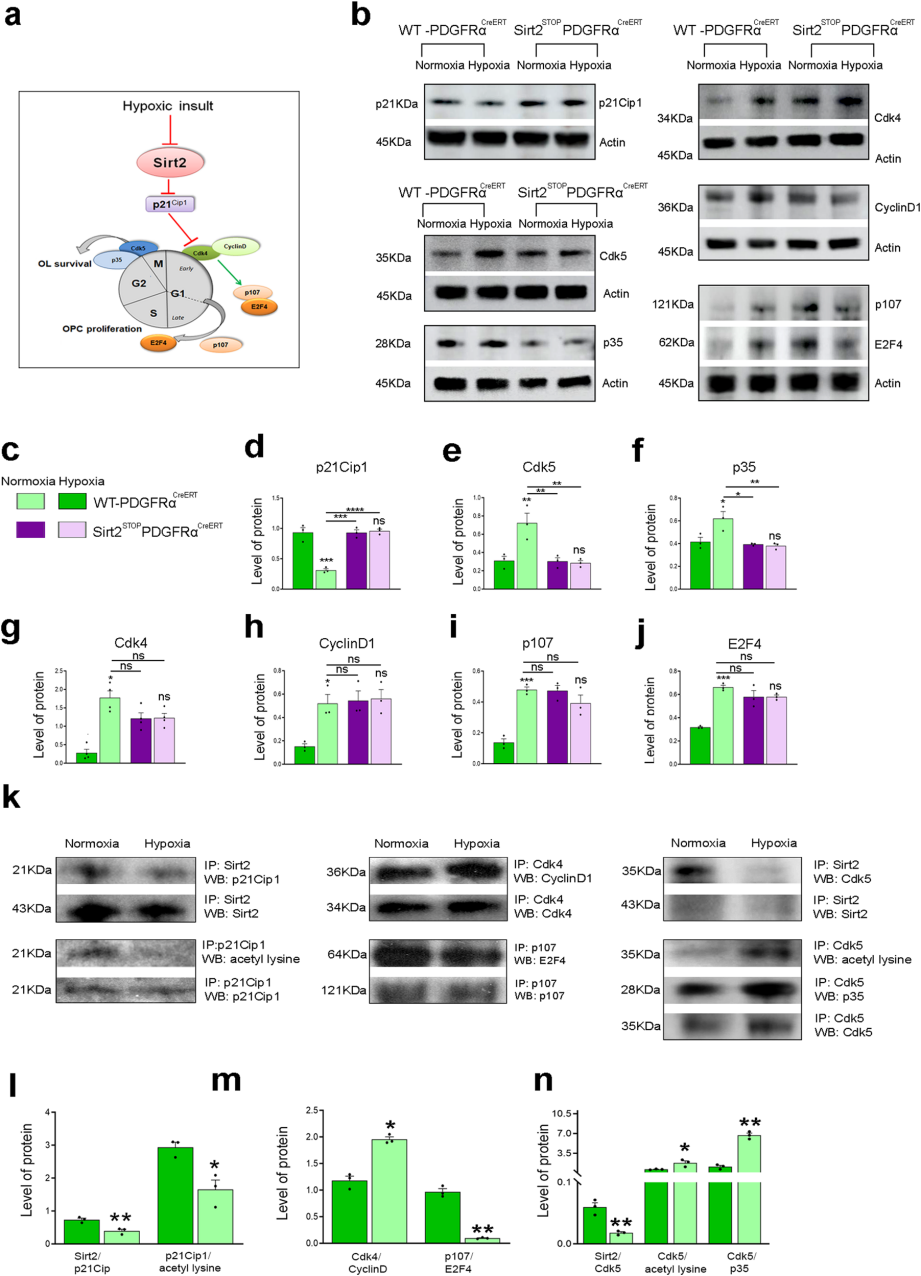
Sirt2 interacts with p21^Cip1^ and Cdk5. **a** Cartoon depicting cell cycle regulation by p21^Cip1^, Cdk4/CyclinD1, and Cdk5/p35. **b** Expression of p21^Cip1^, Cdk5, p35, Cdk4, CyclinD1, p107, and E2F4 in Nx and Hx WM of WT-PDGFRα^CreERT^ and Sirt2^STOP^PDGFRα^CreERT^. **c** Color legend for different transgenic mice in Nx and Hx. **d**–**j** Quantification of protein levels of p21^Cip1^ (WT Nx vs Hx ***p = 0.0001, WT Hx vs Sirt2^STOP^ Nx ***p = 0.0001, WT Hx vs Sirt2^STOP^ Hx ****p < 0.0001) **d**, Cdk5 (WT Nx vs Hx **p = 0.0076, WT Hx vs Sirt2^STOP^ Nx **p = 0.0068, WT Hx vs Sirt2^STOP^ Hx **p = 0.0068) **e**, p35 (WT Nx vs Hx *p = 0.0193, WT Hx vs Sirt2^STOP^ Nx *p = 0.0106, WT Hx vs Sirt2^STOP^ Hx **p = 0.0077) **f**, Cdk4 (*p = 0.0292) **g**, CyclinD1 (*p = 0.0243) **h**, p107 (***p = 0.0005) **i**, and E2F4 (***p = 0.0002) **j** in WT-PDGFRα^CreERT^ and Sirt2^STOP^PDGFRα^CreERT^ mice (*n* = 3 per group, all ANOVA test with Tukey’s multiple comparisons adjustment). Graphs display mean ± SEM values. **k** Co-immunoprecipitation of: Sirt2/p21^Cip1^ and acetyl-lysine p21^Cip1^, Cdk4/CyclinD1 and p107/E2F, Sirt2/Cdk5, acetyl-lysine Cdk5, and Cdk5/p35 complexes from Nx and Hx WM. Protein complexes were identified by their sizes (p21^Cip1^−21kDa, Cdk4-34kDa, Cdk5-35kDa, CyclinD1-36kDa, p35-28kDa, p107-121kDa, E2F4-62kDa, respectively) **l**–**n** Quantification of co-immunoprecipitation results for Sirt2/p21^Cip1^ (**p = 0.0097), acetyl lysine p21^Cip1^ (*p = 0.0190) **l**, Cdk4/cyclinD1 (*p = 0.0138), p107/E2F4 (**p = 0.023) **m**, Sirt2/Cdk5 (**p = 0.0046), acetyl lysine Cdk5 (*p = 0.0349), Cdk5/p35 (**p = 0.0048) **n** (*n* = 3 brains per group, all Student’s *t* tests). Graphs display mean ± SEM values. All statistical tests are two-sided. Source data are provided as a Source Data file.

We then investigated whether Sirt2 regulates a second cyclin inhibitor, p21^Cip1^, which promotes OL differentiation independently of cell cycle withdrawal^31^. A previous study demonstrated that (i) p21^Cip1^ directly inhibits Cdk4, the main regulator of the G1 checkpoint, and (ii) p21^Cip1^ stability depends on its acetylation (Fig. 6a)^34^. We found reduced levels of p21^Cip1^ in Hx WM of WT mice, which were restored in Sirt2^STOP^PDGFRα^CreERT^ mice (Fig. 6b–d). To establish whether Sirt2 regulates OPC exit from the cell cycle through p21^Cip1^, we determined whether Sirt2 directly interacts with p21^Cip1^. Under Nx conditions, a Sirt2/p21^Cip1^ complex was detected by co-immunoprecipitation, and expression of this complex decreased following Hx (Fig. 6k, l). Interestingly, we also found increased deacetylation of p21^Cip1^ after Hx (Fig. 6k, l), indicating reduced stability of p21^Cip1^. Since p21^Cip1^ normally inhibits Cdk4^35,36^, we then assessed Cdk4 activity. Cdk4 expression was significantly increased in WT mice following Hx and was also increased in both Nx and Hx Sirt2^STOP^PDGFRα^CreERT^ mice (Fig. 6b, g). We also analyzed the expression of CyclinD1, p107, and E2F4 proteins, which are all downstream of Cdk4 and promote cellular proliferation. All three proteins displayed the same expression profile as Cdk4—increased expression following Hx and Sirt2 overexpression in OPCs (Fig. 6b, h, i, j). Since Cdk4 is only active when bound with CyclinD1, we also performed a co-immunoprecipitation assay for Cdk4 and CyclinD1. Indeed, we found higher expression of the Cdk4/CyclinD1 complex after Hx, which corresponded with decreased expression of the p107/E2F4 complex (Fig. 6k, m). This indicates that Cdk4/CyclinD1 activity is increased after Hx, resulting in more unbound E2F4 and cell cycle progression. Together, these results suggest that Sirt2 promotes OL differentiation in Nx conditions through its interaction with p21^Cip1^ and that loss of this interaction following Hx may contribute to cell cycle progression past the G1 checkpoint, resulting in increased OPC proliferation.

Finally, we also analyzed whether OPC exit from the cell cycle following the G2/M checkpoint controlled by Cdk5 was also altered following Hx. Cdk5 promotes cell cycle arrest and differentiation through its interaction with p35 and has also been shown to regulate cell survival and apoptosis^37^. We found that both Cdk5 and p35 expression is increased in WT mice following Hx, and interestingly Sirt2 overexpression in OPCs reduced this increase (Fig. 6b, e, f). Furthermore, we found that Sirt2 formed a complex with Cdk5 in Nx conditions, which was strongly downregulated after Hx (Fig. 6k, n). Interestingly, we found that acetylation of Cdk5 increased after Hx, indicating that Sirt2 normally deacetylates Cdk5 (Fig. 6k, n). This corresponded with increased expression of the Cdk5/p35 complex in Hx conditions (Fig. 6k, n). Therefore, Sirt2 also interacts with Cdk5 under Nx conditions, and this interaction is reduced following Hx, potentially contributing to the increased levels of apoptosis observed in Hx WM.

### Nuclear role of Sirt2 in OPCs

**Fig. 7:**
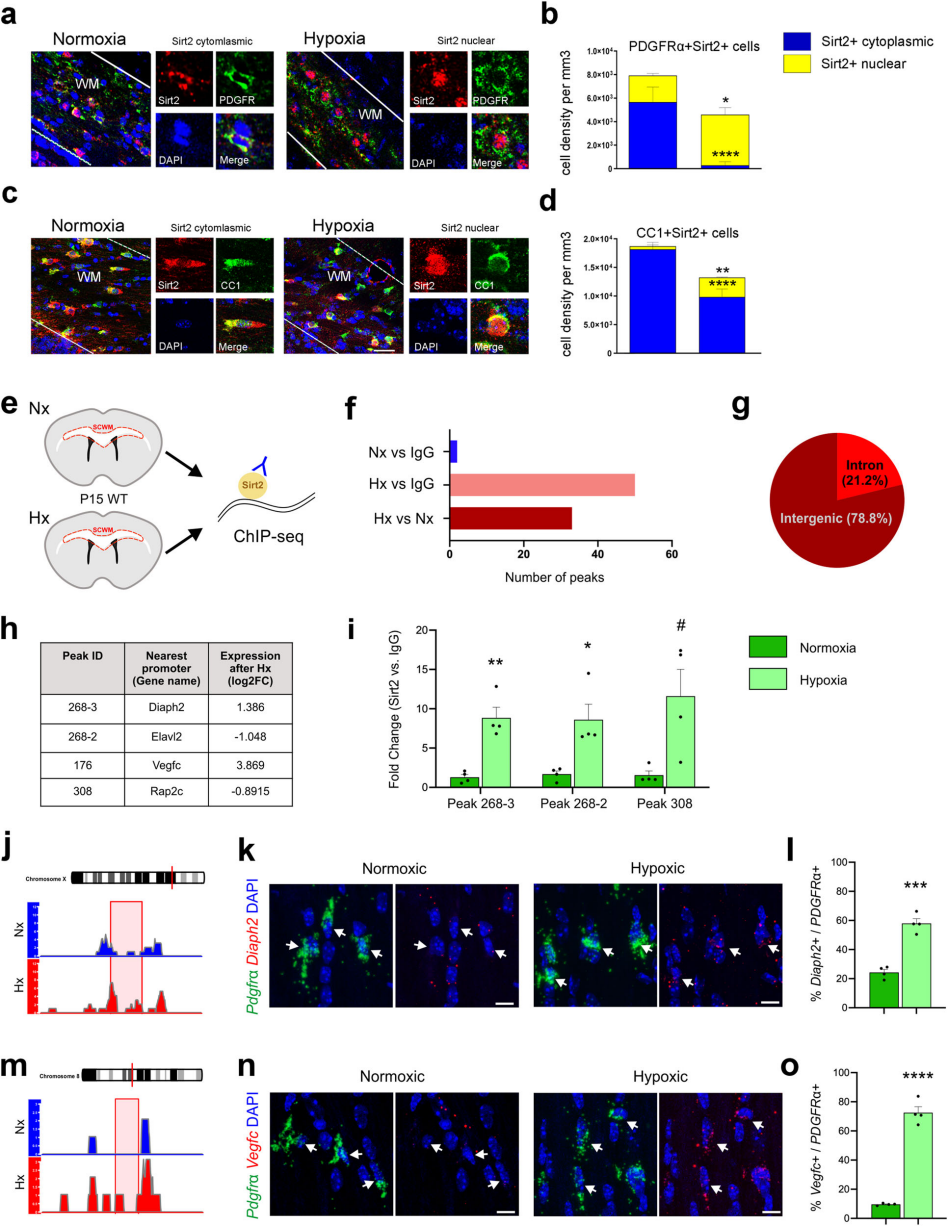
Hx induces nuclear localization of Sirt2 in OPCs. **a**, **c** Coronal sections of subcortical WM from Nx and Hx WT mice at P18. Dotted lines delineate WM, WM-white matter. Scale bar = 100 µm. **b** Quantification of cytoplasmic and nuclear Sirt2 expression in PDGFRα^+^ OPCs (Nx vs Hx: ****p < 0.0001, *p = 0.0152, Student’s *t* test) **d** Quantification of cytoplasmic and nuclear Sirt2 expression in CC1^+^ OLs (Nx vs Hx: **p = 0.0042, ****p < 0.0001, Student’s *t* test). Graphs display mean ± SEM values (*n* = 4 brains per condition). **e** Experimental procedure for Sirt2 ChIP-seq using dissected subcortical white matter (SCWM) from P15 WT mice reared under Nx or Hx conditions. **f** The number of enriched Sirt2 binding peaks identified following comparison of Nx, Hx, and IgG (negative control) samples. **g** The location of Sirt2 peaks enriched after Hx. **h** Sirt2 ChIP-seq data were compared with previously published RNA-seq data from Hx OPCs^39^. Four genes that have nearby Sirt2 binding peaks are also altered in their expression following Hx. **i** QPCR verification of enriched Sirt2 binding at peaks 268-3, 268-2, and 308 in Hx WM, compared to Nx (**p = 0.0017, *p = 0.014, ^#^*p* = 0.059 Student’s *t* test, *n* = 4 Nx and Hx samples). **j** Visualization of Sirt2 binding in genomic region upstream of *Diaph2* gene. Red box highlights enriched binding in Hx. **k** RNAscope analysis of *Diaph2* expression in *Pdgfrα*^+^ OPCs (arrows) in the WM of Nx and Hx WT mice at P22. Scale bars = 10 µm. **l** Quantification of the percentage of *Diaph2*^+^ OPCs in the WM of Nx and Hx WT mice at P22 (***p = 0.0001, *n* = 4 Nx and Hx mice, Student’s *t* test). **m** Visualization of Sirt2 binding in the genomic region upstream of *Vegfc* gene. Red box highlights enriched binding in Hx. **n** RNAscope analysis of *Vegfc* expression in *Pdgfrα*^+^ OPCs (arrows) in the WM of Nx and Hx WT mice at P22. Scale bars = 10 µm. **o** Quantification of the percentage of *Vegfc*^+^ OPCs in the WM of Nx and Hx WT mice at P22 (****p < 0.0001, Student’s *t* test, *n* = 4 Nx and Hx mice). Graphs display mean ± SEM values. All statistical tests are two-sided. Source data are provided as a Source Data file. The schematic in **e** was created using CorelDraw 2018 software (version 20.1.0.708).

Sirt2 is predominantly cytoplasmic during the entire cell cycle except the G2/M transition, when it translocates to the nucleus as a result of active shuttling between these two compartments^20,38^. To investigate a cytoplasmic-nuclear translocation of Sirt2 in WM cells under normal and pathological conditions, we determined the subcellular localization of Sirt2 in PDGFRα^+^ OPCs and mature CC1^+^ OLs. We found that under the Nx condition Sirt2 was localized to the cytoplasm in the majority of Sirt2^+^ OPCs (Fig. 7a, b). However, in Hx Sirt2 was localized to the nucleus in the majority of Sirt2^+^ OPCs (Fig. 7a, b). In contrast, virtually almost all mature CC1^+^Sirt2^+^ OLs expressed Sirt2 in the cytoplasm in Nx, and Hx induced its translocation into the nucleus in only a small number of OLs (Fig. 7c, d). Therefore, Hx induces a significant change in Sirt2 subcellular location specifically within OPCs.

In order to define the role of nuclear Sirt2 in OPCs and OLs, we performed chromatin immunoprecipitation (ChIP) for Sirt2 using dissected WM samples from Nx and Hx mice. Since there is not a commercially validated anti-Sirt2 antibody for ChIP, five different antibodies (# sc211033, # sc223534, # sc51023, # sc67299, and # S8447) were compared for their ability to detect Sirt2 protein in both Western blots of dissected WM tissue and immunocytochemistry on cultured Nx and Hx cells. We determined that anti-Sirt2 antibody (# sc211033) was the best candidate for ChIP, based on its specificity in Western blots and nuclear staining in cultured cells (Supp. Fig. 9a). Other antibodies either did not detect the appropriately sized band for Sirt2 (43 kD) or resulted in mostly cytoplasmic staining (Supp. Fig. 9b). We performed next-generation sequencing following ChIP (ChIP-seq) to identify potential genomic targets of nuclear Sirt2 in both Nx and Hx WM (Fig. 7e). Interestingly, we only identified two enriched peaks in Nx WM, compared to the IgG negative control (Fig. 7f). Both peaks occurred upstream of the microRNA mir-101c gene (Supp. Table 2). In comparison, we detected 50 enriched peaks in Hx WM, compared to IgG negative control (Fig. 7f, Supp. Table 2). When we directly compared Nx and Hx WM samples to each other, we identified 33 enriched peaks under Hx conditions (Fig. 7f, Supp. Table 2), indicating that there is increased DNA-binding activity of Sirt2 following Hx. We performed additional annotation and analysis of these 33 peaks, finding that the majority are located in intergenic regions of the genome (Fig. 7g). We identified the nearest promoter for each Hx-upregulated peak and searched our previously published OPC RNA-seq dataset^39^ to determine whether any Sirt2 bound genes displayed changes in gene expression following Hx. We found that 4 genes (*Diaph2*, *Elavl2*, *Vegfc*, and *Rap2c*) were present in both datasets (Fig. 7h), indicating that Sirt2 nuclear translocation in OPCs may underly specific alterations in gene expression following Hx. To verify these ChIP-seq results we performed qPCR on additional Nx and Hx samples for three of the identified peaks (268-3, 268-2, and 308), and found increased enrichment for these genomic regions in our Sirt2 ChIP samples, over IgG negative controls (Fig. 7i). In addition, we visualized the Sirt2 binding peaks in Nx and Hx samples for the regions upstream of *Diaph2* and *Vegfc*, confirming increased binding activity following Hx (Fig. 7j, m). Finally, we confirmed with RNAscope that both *Diaph2* and *Vegfc* increase in expression within WM OPCs following Hx (Fig. 7k, l, n, o). Together, these results identify an additional nuclear role of Sirt2 that is specific to Hx WM injury, highlighting the complexity of Sirt2’s cellular functions.

### Sirt1 inactivation induces an increase in Sirt2^+^ OLs in WM

**Fig. 8:**
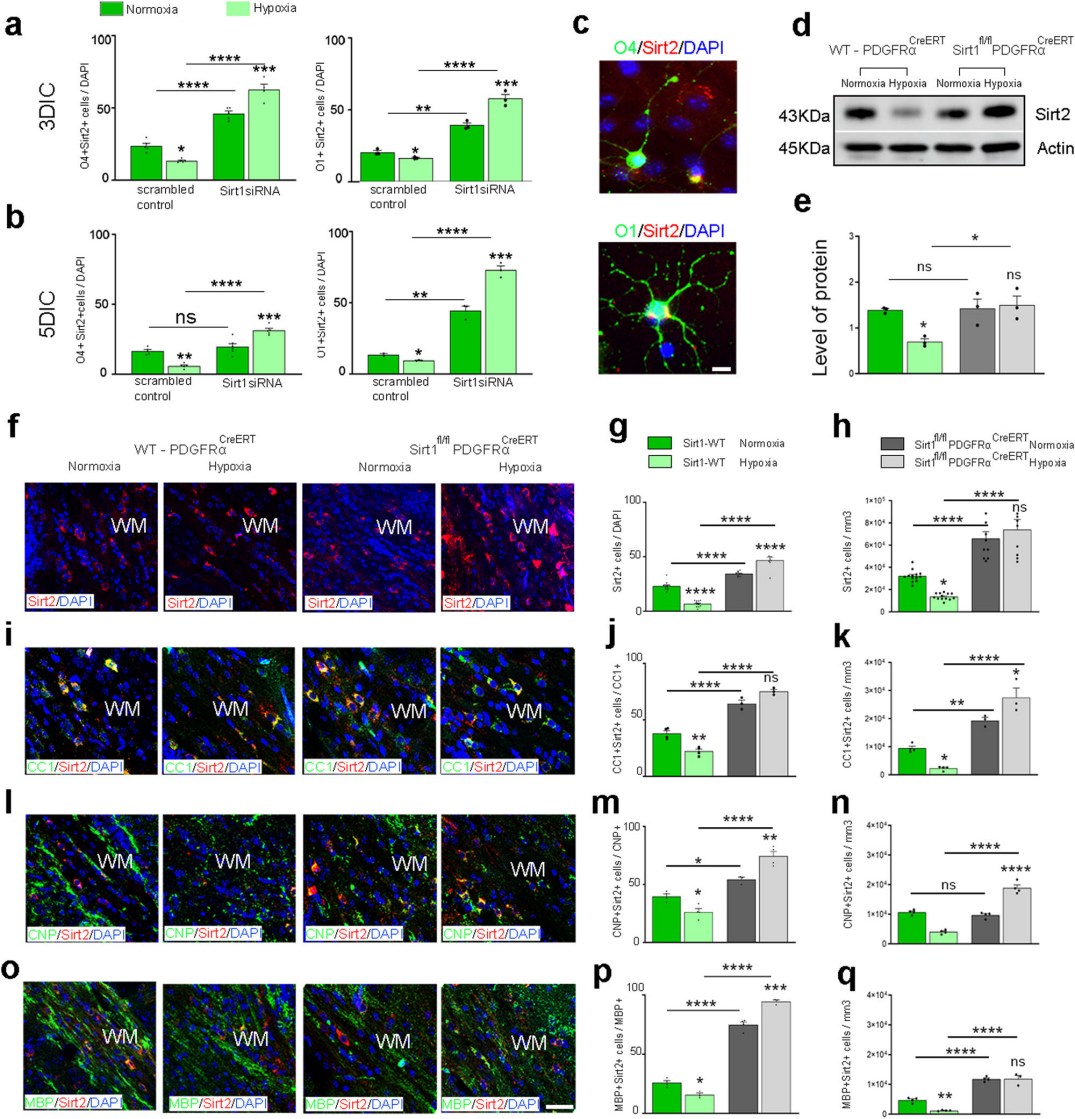
Increased differentiation of Sirt2^+^ OLs in the absence of Sirt1. **a**, **b** Quantification of cultured WM cells from Nx and Hx mice transfected with scrambled control or Sirt1 siRNA and cultured for 3 days **a** (O4: control *p = 0.0121, Sirt1 siRNA ***p = 0.0003, Nx control vs Nx siRNA ****p < 0.0001, Hx control vs Hx siRNA ****p < 0.0001; O1: control *p = 0.0440, Sirt1 siRNA ***p = 0.0004, Nx control vs Nx siRNA **p = 0.0024, Hx control vs Hx siRNA ****p < 0.0001) and 5 days in culture (DIC) **b** (O4: control **p = 0.0013, Sirt1 siRNA ***p = 0.0003, Nx control vs Nx siRNA ns *p* = 0.5243, Hx control vs Hx siRNA ****p < 0.0001; O1: control *p = 0.0265, Sirt1 siRNA ***p = 0.0003, Nx control vs Nx siRNA **p = 0.0032, Hx control vs Hx siRNA ****p < 0.0001, ANOVA with Tukey’s multiple comparisons adjustment) (*n* = 3 mice per group). **c** Representative images of O4^+^Sirt2^+^ and O1^+^Sirt2^+^ cells in Nx cultures. Scale bar = 50 µm. **d** Representative western blot from Nx and Hx WM lysates from WT-PDGFRα and Sirt1^fl/fl^PDGFRα^CreERT^ mice for Sirt2 expression (*n* = 3 per group). Molecular weight for Sirt2–43 KD **e**) Quantification of Sirt2 expression in Nx and Hx WM of WT-PDGFRα^CreERT^ (green) and Sirt1^fl/fl^PDGFRα^CreERT^ (gray) mice. (WT Nx vs Hx *p = 0.0390, WT Hx vs Sirt1^fl/fl^PDGFRα^CreERT^ Hx *p = 0.0184, ANOVA with Tukey’s multiple comparisons adjustment). **f**, **i**, **l**, **o** Coronal sections of subcortical WM from WT-PDGFRα and Sirt1^fl/fl^PDGFRα^CreERT^ mice, after Nx and Hx, showing Sirt2^+^ **f**, CC1^+^Sirt2^+^ **i**, CNP^+^Sirt2^+^ **l**, and MBP^+^Sirt2^+^ **o** cells. WM-white matter. Scale bar = 100 µm. **g**, **j**, **m**, **p** Quantification of the percentage of WM cells that express Sirt2 (all ****p < 0.0001, ANOVA with Tukey’s multiple comparisons adjustment) **g**, CC1^+^Sirt2^+^ (WT: Nx vs Hx **p < 0.0020, Sirt1^fl/fl^PDGFRα^CreERT^: Nx vs Hx ns *p* = 0.0517, WT Nx vs Sirt1^fl/fl^PDGFRα^CreERT^ Nx ****p < 0.0001, WT Hx vs Sirt1^fl/fl^PDGFRα^CreERT^ Hx ****p < 0.0001, ANOVA with Tukey’s multiple comparisons adjustment) **j**, CNP^+^Sirt2^+^ (WT: Nx vs Hx *p = 0.0351, Sirt1^fl/fl^PDGFRα^CreERT^: Nx vs Hx **p = 0.0043, WT Nx vs Sirt1^fl/fl^PDGFRα^CreERT^ Nx *p = 0.0370, WT Hx vs Sirt1^fl/fl^PDGFRα^CreERT^ Hx ****p < 0.0001, ANOVA with Tukey’s multiple comparisons adjustment) **m**, and MBP^+^Sirt2^+^ (WT: Nx vs Hx *p = 0.0204, Sirt1^fl/fl^PDGFRα^CreERT^: Nx vs Hx ***p = 0.0002, WT Nx vs Sirt1^fl/fl^PDGFRα^CreERT^ Nx ****p < 0.0001, WT Hx vs Sirt1^fl/fl^PDGFRα^CreERT^ Hx ****p < 0.0001 **p**, ANOVA with Tukey’s multiple comparisons adjustment) after Nx and Hx in WT and Sirt1^fl/fl^PDGFRα^CreERT^ mice. **h**, **k**, **n**, **r** Quantification of the total density of WM cells that express Sirt2^+^ (WT: Nx vs Hx *p < 0.0188, Sirt1^fl/fl^PDGFRα^CreERT^: Nx vs Hx ns *p* = 0.6630, WT Nx vs Sirt1^fl/fl^PDGFRα^CreERT^ Nx ****p < 0.0001, WT Hx vs Sirt1^fl/fl^PDGFRα^CreERT^ Hx ****p < 0.0001, ANOVA with Tukey’s multiple comparisons adjustment) **h**, CC1^+^Sirt2^+^ (WT: Nx vs Hx *p = 0.0464, Sirt1^fl/fl^PDGFRα^CreERT^: Nx vs Hx *p = 0.0104, WT Nx vs Sirt1^fl/fl^PDGFRα^CreERT^ Nx **p = 0.0055, WT Hx vs Sirt1^fl/fl^PDGFRα^CreERT^ Hx ****p < 0.0001, ANOVA with Tukey’s multiple comparisons adjustment) **k**, CNP^+^Sirt2^+^ (WT: Nx vs Hx ****p < 0.0001, Sirt1^fl/fl^PDGFRα^CreERT^: Nx vs Hx ****p < 0.0001, WT Nx vs Sirt1^fl/fl^PDGFRα^CreERT^ Nx ns *p* = 0.6821, WT Hx vs Sirt1^fl/fl^PDGFRα^CreERT^ Hx ****p < 0.0001, ANOVA with Tukey’s multiple comparisons adjustment) **n**, and MBP^+^Sirt2^+^ (WT: Nx vs Hx **p = 0.0027, Sirt1^fl/fl^PDGFRα^CreERT^: Nx vs Hx ns *p* = 0.9997, WT Nx vs Sirt1^fl/fl^PDGFRα^CreERT^ Nx ****p < 0.0001, WT Hx vs Sirt1^fl/fl^PDGFRα^CreERT^ Hx ****p < 0.0001, ANOVA with Tukey’s multiple comparisons adjustment) **q** after Nx and Hx in WT and Sirt1^fl/fl^PDGFRα^CreERT^ mice. Graphs display mean ± SEM values (*n* = 3 animals per group). All statistical tests are two-sided. Source data are provided as a Source Data file.

Previously, we found that Sirt1 regulates OPC proliferation in basal and Hx conditions, and inactivation of Sirt1 promotes OL differentiation^16^. The results from this study indicate that Sirt2 promotes OL differentiation in both Nx and Hx WM. Therefore, we hypothesized that OL development is strongly dependent on the activities of both Sirt1 and Sirt2, regulating a balance between two crucial developmental processes—OPC proliferation and OL maturation—both in normal WM and in recovery after Hx. To examine this, we investigated the impact of Sirt1 knockdown on Sirt2 expression in cultured OL lineage cells from Nx and Hx WM. In scrambled control cultures, significant reduction in the numbers of Sirt2^+^O1^+^ and Sirt2^+^O4^+^ cells were observed in Hx cells compared to Nx cells, at both 3 and 5 days in culture (DIC) (Fig. 8a–c). However, significantly higher percentages of both O1^+^ and O4^+^ OLs expressed Sirt2 after Sirt1 knockdown, under both Nx and Hx conditions (Fig. 8a–c). We then confirmed these results in vivo using Sirt1^fl/fl^PDGFRα^CreERT^ transgenic mice, in which Sirt1 was ablated in PDGFRα^+^ OPCs after tamoxifen injection at P11 and P12. First, we determined via western blot that Sirt1 ablation from OPCs increased Sirt2 protein levels in both Nx and Hx WM (Fig. 8d, e). We then analyzed several mature OL populations in vivo. As expected, Hx induced a reduction in the number of CC1^+^Sirt2^+^, CNP^+^Sirt2^+^, and MBP^+^Sirt2^+^ OLs in WT mice (Fig. 8f–q). However, Sirt1 ablation increased all these cell populations in both Nx and Hx WM (Fig. 8f–q). Interestingly, Sirt1 expression in OL lineage cells was not changed following either Sirt2 knockdown or overexpression in vivo (Supp. Fig. 10a–c). Together, these results indicate that sequential downregulation of Sirt1 and upregulation of Sirt2 levels/activity are required for crucial OL lineage transition. Furthermore, our findings also indicate that Sirt1 downregulation may be required for upregulation of Sirt2 and OL maturation in the developing WM.

## Discussion

With the occurrence of premature births rising internationally, it is imperative that we obtain a detailed understanding of the molecular and cellular changes that underly WM damage commonly seen in very premature infants. Intrinsic and epigenetic regulators of OL development are of particular interest as potential targets for promoting endogenous recovery from OPCs following neonatal injury. Sirt2’s expression in brain OLs was first reported over a decade ago^19^; and yet, the molecular and cellular roles of Sirt2 in OL development and regeneration in vivo have yet to be investigated. Furthermore, the majority of publications focus on Sirt2’s deacetylation of alpha-tubulin in OLs^40,41^, despite evidence that Sirt2 regulates a wide range of cellular processes in other cell types^21,42,43^. To obtain a more complete understanding of Sirt2’s cellular roles, we explored these unknown questions in the context of OL development and injury in vivo using an established Hx model of neonatal injury. Importantly, this work complements our previous study of Sirt1 function in WM development and repair^16^, providing a more complete and integrated picture of how sirtuins are critical intrinsic regulators of OL biology.

In this study we find that Sirt2 expression in mature OLs is strongly downregulated following Hx – both in the mouse model of neonatal Hx, as well as in preterm human tissue samples, indicating that our mouse model recapitulates cellular phenotypes of human neonatal injury. We show that Sirt2 promotes OL differentiation during both normal WM development and after Hx, which confirms and extends previous in vitro studies. Interestingly, we find that Sirt2 overexpression in PDGFRα-expressing OPCs, but not mature OLs, is able to restore OL populations after Hx through enhanced OPC proliferation *and* protection from apoptosis. To our knowledge, our study is the first to report the role of Sirt2 in OPC cell survival. Other studies have described an impact of Sirt2 inhibition on endothelial cell survival^44^ and cancer cell survival^45–47^, indicating that there may be a conserved role of Sirt2 in preventing apoptosis. Together, these results illustrate that Sirt2 is an appropriate molecular target for promoting oligodendrogenesis following injury. Importantly, the lack of rescue of OL populations following Sirt2 overexpression in PLP^+^ cells suggests that the timing of Sirt2 expression during OL lineage maturation is critical for this regenerative response.

The molecular mechanisms regulating the number of proliferating OPCs and differentiating OLs are important for both normal development and processes of repair in pathological conditions. Cdk-cyclin complexes that orchestrate cell cycle progression are regulated via their association with inhibitory proteins, including the Kip/Cip family (p27^Kip1^ and p21^Cip1^). However, the full extent of these protein interactions in OL development and regeneration remains to be determined. We previously found that recovery of OL populations after neonatal Hx requires p27^Kip1^ expression, as knockdown of p27^Kip1^ exacerbated the delay in OL differentiation induced by Hx insult^7^. p27^Kip1^ activity is mainly controlled by post-translational modifications, particularly phosphorylation, ubiquitination, and acetylation^48^. We find that Sirt2 interacts with p27^Kip1^ in WM cells under Nx conditions, indicating that Sirt2 may regulate its acetylation level and activity. Interestingly, expression of the Sirt2/p27^Kip1^ complex increases after Hx, corresponding with an increase in p27^Kip1^ deacetylation. Therefore, Sirt2’s increased association with p27^Kip1^ likely promotes the subsequent degradation and loss of p27^Kip1^ expression following Hx. However, because protein degradation is dependent on the balance between deacetylation and ubiquitination^49^, we cannot exclude the possibility that decreased p27^Kip1^ expression after Hx occurs also via the ubiquitination-proteasome pathway.

We previously also observed decreased FoxO transcription factor expression in Hx WM^7^. FoxO transcription factors regulate a variety of cellular processes, including cell cycle progression, differentiation, and apoptosis^50–52^. Although other studies have reported changes in FoxO activity in response to oxidative stress and after Hx/reoxygenation injury^53–55^, the molecular mechanisms remain unknown. Sirtuins are known to deacetylate FoxOs directly, as well as regulate adipocyte differentiation through FoxO1 deacetylation^42,56^. In our Hx injury model, we observe increased formation of a Sirt2/FoxO1 complex and increased deacetylation of FoxO1, compared to Nx conditions. Since, FoxO1 is a known promoter of p27^Kip1^ expression^57^, increased FoxO1 degradation induced by Sirt2 deacetylation likely leads to decreased p27^Kip1^ expression. Therefore, it appears that Sirt2 both directly and indirectly (via FoxO1) promotes p27^Kip1^ downregulation following Hx. Since p27^Kip1^ is the main player in OL differentiation^29,30,58^, its reduction is likely responsible, at least in part, for insufficient OL differentiation after Hx. A fascinating question that remains is how Hx promotes increased interaction of Sirt2 with p27^Kip1^ and FoxO1.

Hx induces OPC proliferation^7^, partly through increased Sirt1 expression^16^. Interestingly, Sirt2 overexpression in PDGFRα-expressing OPCs also elevates proliferation both under Nx and Hx conditions, possibly mimicking Sirt1’s pro-proliferative activity. Another cell cycle inhibitor, p21^Cip1^ is required for OPC proliferation and differentiation in vitro^31^ and can act as a positive regulator of cell cycle entry^59^. We found that Sirt2/p21^Cip1^ complex expression decreases after Hx, which paradoxically corresponds with increased deacetylation of p21^Cip1^. This suggests that a different deacetylase enzyme associates with p21^Cip1^ following Hx (due to the loss of interaction with Sirt2). One possible candidate is Sirt1, which we will examine in future work. Ultimately, this results in increased Cdk4/cyclin D interaction, which then alters the activity of pocket proteins, including p107 associated with E2F4 transcription factor^60^. Since p21^Cip1^ may interact directly or indirectly with different transcription factors such as Myc, E2Fs, or STAT3 and regulate their activity^61–63^, it cannot be excluded that an indirect regulation of E2F4 transcription factor by p21^Cip1^ occurs in Hx WM. However, our results show higher levels of the unbound E2F4 transcription factor, suggesting that enhanced OPC proliferation after Hx is at least partly mediated by alterations in this p21^Cip1^-Cdk4/cyclin D/E2F4 pathway induced by loss of Sirt2/p21^Cip1^ interaction.

Particularly interesting is also Sirt2’s interaction with Cdk5. While Cdk5 has been primarily described as a crucial regulator of neuronal migration^64^, it also regulates cell survival and apoptosis^37,65^. Interestingly, Cdk5 acts as both an inhibitor and inducer of neuronal cell death, with this switch occurring via the cleavage of the Cdk5 activator p35 into p25^66,67^. Furthermore, enhanced Cdk5 and p35 immunoreactivity were observed in apoptotic cells following ischemia^68^. In our study, we observe decreased interaction of Sirt2/Cdk5 following Hx, resulting in increased acetylation of Cdk5 and increased interaction of Cdk5/p35. This increase may be partly responsible for the increase in apoptosis observed following Hx^7^, and may explain why Sirt2 overexpression in OPCs prevents apoptosis. Future work will examine this pathway in more detail to better understand Cdk5 signaling in OLs. Interestingly, Cdk5/p35 has also been shown to phosphorylate Sirt2, inhibiting Sirt2’s catalytic activity^29^, indicating that there may be some feed-forward mechanisms also at play.

Finally, the complexity of sirtuin activities is also dependent on their subcellular localization and translocation under various pathologies. A previous study in yeast found that Sirt2 is cytoplasmic during the entire cell cycle, except in the G2/M transition phase, when it translocates to the nucleus and deacetylates histone H4 at lysine 16, leading to chromatin modification^38^. We observe an increase in nuclear Sirt2 expression following Hx, but only in OPCs. Importantly, we find that Sirt2 binds more genomic targets in Hx WM, compared to Nx. Several of these targets are in close proximity to genes whose expression is altered following Hx, indicating that nuclear Sirt2 translocation has functional outcomes and may explain some of the previously reported molecular changes that occur in the OL lineage in response to Hx^39^. One of these genes is *Diaph2*, which has been shown to nucleate actin, bind and stabilize microtubules, and be involved in cell division^69^. This complements Sirt2’s known activity as a tubulin deacetylase^70^, suggesting that Sirt2 may regulate microtubule dynamics via both direct and indirect mechanisms. A second gene is *Vegfc*, which was previously found to be upregulated in a rat model of Hx/ischemia and promotes OPC proliferation in vitro^71^. Therefore, in addition to its cytoplasmic protein interactions, Sirt2 also acquires a nuclear function in OPCs that contributes to the molecular and cellular alterations observed in Hx WM.

Sirt1 and Sirt2 regulate numerous cellular processes under normal and pathological conditions^14,16,72–74^. Opposing effects of sirtuins on neuronal survival has been previously described, with Sirt1 protecting against neuronal death, while Sirt2 induced apoptosis^75^. Our developmental studies revealed a reciprocal pattern of expression of these two sirtuins in WM. This pattern is consistent with developmental trajectories of OPC proliferation and differentiation, suggesting a functional interplay between Sirt1 and Sirt2. Previously, we reported that Sirt1 ablation reduces OPC proliferation and increases OL differentiation^16^, whereas this current study shows that Sirt2 ablation reduces OL differentiation. Furthermore, we find that Sirt1 knockdown promotes increased expression of Sirt2^+^ OLs, suggesting Sirt1 actively represses Sirt2 expression under normal conditions. In addition, the subcellular localizations of both Sirt1 and Sirt2 in OL lineage cells complement each other. In normal conditions, nuclear Sirt1 is localized in OPCs^16^ and cytoplasmic Sirt2 is present mainly in OLs. Following Hx, Sirt1 translocates to the cytoplasm^16^ and Sirt2 translocates to the nucleus in OPCs. The mechanisms underlying these changes in cellular localization remain unknown, but as shown through our ChIP-Seq data, likely result in epigenetic and gene expression changes. Sirt1 and Sirt2 share some common cytoplasmic targets, such as FoxO1 and FoxO3a^76^, and are both known to deacetylate lysine 16 of histone H4^11,38^, indicating there may be some functional compensation. Overall, these results highlight that Sirt1 and Sirt2 coordinate complex networks of protein and DNA interactions on individual and combinatorial levels that are essential for correct WM development. Furthermore, both proteins are distinctly altered by Hx, contributing to the cellular pathology. Interestingly, Sirt1 and Sirt2 have also been found to play important roles in neurodegenerative disorders such as Alzheimer’s, Parkinson’s, and Huntington’s disease by exhibiting opposing effects in which activation of Sirt1 and inhibition of Sirt2 have neuroprotective roles^72^. Our work, therefore, has important implications for WM damage and repair in neurodegenerative disease, which is of high interest to the neuroscience field.

In conclusion, sirtuins are deacetylases that are shown to have therapeutic potential in various diseases, including aging, neurodegenerative disorders, cardiovascular disease, and cancer. Here, the link between Sirt2 and OL development provides a new mechanism for WM injury. Using a mouse model of chronic neonatal Hx, which recapitulates the WM injury in preterm neonates^77^, we defined a crucial role of Sirt2 in OL differentiation and repair. Overexpression of Sirt2 in OPCs, but not mature OLs, partially rescues Hx-induced injury, a major insult in preterm born neonates, by targeting OL maturation and pro-survival signaling pathways. Sirt2 reduction in preterm human WM likely contributes to the arrest in pre-OL maturation and myelination failure seen in the extremely low gestational age neonates (ELGANs)^78^. By this action, Sirt2 may be an opportunity to capture the early and tight window of opportunity for intervention prior to chronic WM injury and astrogliosis^78^. Given that Sirt2 modifies multiple early signaling pathways, future efforts should focus on which protein and/or genomic interactions and the right time points are most significant in promoting repair. Overall, our studies open a new avenue for Sirt2 as a therapeutic target for WM injury in preterm born neonates affected by Hx.

## Methods

All methods were performed in accordance with the guidelines and regulations of Children’s National Hospital and Children’s National Research Institute. All animal procedures were approved by the Institutional Animal Care and Use Committee (IACUC) of the Children’s National Hospital (protocol #30473).

### Animals

WT (C57BL/6), Sirt2^STOP^, Sirt1^fl/fl^, Sirt2^fl/fl^, PDGFRα^CreERT^, PLP^CreERT^ (Jackson Laboratory cat #003548, 029604, 029603, 030835, 032770, 005975), CD1 (Charles River Crl:CD1(ICR)), and CNP-EGFP^79^ mice were maintained in the animal facility of the Children’s National Hospital according to the Institutional Animal Care and Use Committee and the National Institutes of Health guidelines. To obtain mice in which *Sirt1* or *Sirt2* was conditionally ablated in OPCs, we crossed Sirt1^fl/fl^ or Sirt2^fl/fl^ mice (where loxP sites flanked exon 4 of the *Sirt1* gene, or exons 5–7 of the *Sirt2* gene) with PDGFRα^CreERT^ transgenic mice, which express the tamoxifen-inducible form of *Cre* in OPCs expressing PDGFRα. To obtain mice in which *Sirt2* was conditionally overexpressed in PDGFRα^+^ OPCs or PLP^+^ mature OLs, we crossed Sirt2^STOP^ mice with PDGFRα ^CreERT^ or PLP^CreERT^ transgenic mice, respectively. Both male and female mice were analyzed for all experiments. Mice were maintained in the animal facility under a 12 h dark–light cycle, and constant temperature (20–26 °C) and humidity maintenance (40–60%).

### Hypoxia paradigm

Mice were exposed to 9.5–10.5% oxygen concentration in a Hx chamber on postnatal day 3 (P3). To optimize nutrition during Hx, transgenic pups were housed in the chamber with a CD1 foster mother and her pups. At P11, mice were removed from the chamber and transferred to a room with Nx air conditions, remaining under the foster mother’s care to minimize stress. For all strains, exposure to Hx lasted 8 consecutive days (P3–P11). For all in vivo loss-of-function and gain-of-function experiments, transgenic mice were injected with tamoxifen intraperitoneally twice—at P11 and P12 (50 mg/kg of body weight each)—and then sacrificed at P18.

### Human tissue histology (H&E)

Neuropathological assessment of human brain tissue was performed as previously described^80^. Briefly, the human brain specimens were in the form of formalin-fixed and paraffin-embedded tissue blocks. Sections were obtained (10 μm thickness) and slides were rehydrated with xylene and stained with H&E. Cells were evaluated and counted using morphological criteria. Features of ischemic injury included nuclear hyperchromasia, nuclear pyknosis, cytoplasmic eosinophilia, cytoplasmic shrinkage, cytoplasmic micro-vacuolation, and cell homogenization. We used an Olympus BX43 microscope using 2×, 20×, and 60× objectives to image the corpus callosum tissue. Images were captured using CellSense 4.1 software.

### Human tissue—immunohistochemistry and cell counting

A total of eight human subjects were enrolled from NIH Neurobiobank and Children’s National Pathology Department. The experiments with human brains were approved by the Institutional Review Board at Children’s National Hospital (IRB#00011850), were considered discarded tissues, and no consent was required. All methods were performed in accordance with the guidelines and regulations of Children’s National Hospital and Children’s National Research Institute. A total of 8 autopsy cases of infants—4 with diagnosis of preterm birth less than 32 weeks of gestation and 4 infants born at term (>37 weeks of gestation)—were identified. The human brain specimens were in the form of formalin-fixed and paraffin-embedded tissue blocks. Sections were obtained (10 μm thickness) and were processed for immunohistochemical staining with Olig2 (1:200, R&D #AF2418) and Sirt2 antibodies (1:200, Abcam #211033). Following deparaffinization, antigen retrieval was performed for 10 min in citrate buffer (pH 6) in the microwave. Slides were then rinsed in PBS with 0.2% Triton-X and incubated in blocking reagent (PerkinElmer; TSA kit) for 1 h. Slides were incubated in primary antibodies at 4 °C for 48 h, followed by incubation with species-specific secondary antibodies for 2 h at room temperature. Amplification of Olig2 signal was performed using a Biotin-SP-conjugated secondary antibody (1:500, Jackson, 705-065-147), followed by incubation in streptavidin-HRP (1:2000, Perkin Elmer, NEL750) for 30 min at room temperature and TSA-FITC for 5–10 min at room temperature. Z-stacks of 1 µm-thick optical sections through the entire slice were captured using a confocal microscope (Leica, LAS X software) and collapsed along *z* axis before cell counting in FIJI. Measurements were taken from five images per tissue sample. The results are presented as mean ± SEM.

### Mouse tissue—immunohistochemistry and cell counting

Immunohistochemistry was performed on floating sections using antibodies against the following antigens: NG2 (1:250, Chemicon, AB5320), GFAP (1:500, Chemicon, MAB3402), Olig2 (1:200, Abcam, ab33437), CC1 (1:500, CalBiochem, OP80), Sirt2 (1:200, ABCAM, ab67299), Iba1 (1:200, WAKO, 019-19741), PDGFRα (1:250, BD Bioscience 558774), CNP (1:100, Biolegend, 836404), MBP (1:200, Biolegend, 808402). All antibody dilutions were as previously described^7,16^. Sections were incubated overnight at 4 °C in primary antibodies diluted in 0.1 M PBS (pH7.4), containing 0.1% Triton and 5% normal goat serum. Appropriate secondary antibodies were used as follow: TRITC-conjugated AffiniPure Goat Anti-Mouse IgG (H + L), FITC-conjugated AffiniPure Goat Anti-Rabbit IgG, TRITC-conjugated AffiniPure Goat Anti-Mouse IgM (all 1:200, 115-025-146, 111-095-008, 115-025-020, respectively, all from Jackson ImmunoResearch). Sections were incubated with secondary antibodies for 1 h at room temperature and then mounted. Z-stacks of 1µm-thick optical sections through the entire slice were captured using a confocal microscope (Zeiss LSM or Zen 2.3 software and Leica, LAS X software) and collapsed along *z* axis before cell counting. Measurements were taken from 7–12 tissue sections obtained from 3–4 mice in each group. The results are presented as mean ± SEM.

### Cell cultures

WM was dissected from 300 µm-thick brain sections obtained from Nx and Hx mice at P18, and digested in Hanks’ Balanced Salt Solution (Gibco, 14170-161) containing papain (13 units/ml, Sigma, T4762), DNAse (5 units/ml, Sigma, D5427) and trypsin (Sigma, T4799) for 30 min at 37 °C. Cells were dissociated by trituration and resuspended in Hanks’ Buffer containing 1 M HEPES (BioSource, P305), 15% sucrose, and Penicilin/Streptomycin. Cells were then plated onto poly-L-lysine-coated dishes at a density of 10 cells/µl and cultured for 10 days in D-MEM/F12 medium (Gibco, 11330-032), supplemented with 1% N2 and 1% B27 (Gibco, 17502-048, 17504-044), and with 20 ng/ml EGF and 10 ng/ml bFGF (Upstate Biotechnology, 01-407, 01-114). To immunolabel differentiated cells, standard protocols were used^7,16^ with primary antibodies against GalC (1:200, Galactocerebroside; Abcam ab142) and Olig2 (1:200, Abcam, ab9610).

### FACS

To purify mature OLs, WM was dissected from Nx and Hx CNP-EGFP mice at P18 and dissociated into single cells as described above. CNP-EGFP^+^ OLs were then FACS-purified as previously described^7^ (Influx, Cytopeia, Seattle, WA). After washing with DMEM/F12 medium, CNP-EGFP^+^ cells were plated in 24-well plates coated with poly-l-lysine at the density of 10 cells/μl and cultured in Stem Cell Medium (Stem Cell Technology, Inc.) containing 20 ng/ml EGF and 10 ng/ml bFGF (Upstate Biotechnology). In order to isolate NG2^+^ OPCs, single-cell suspensions obtained from Nx and Hx CNP-EGFP mice were incubated with anti-NG2 antibody (1:1000; Chemicon) for 1 hr at 4 °C. Cells were then washed twice in D-MEM/F12 medium and incubated with Alexa 647 (Jackson Immunoresearch) for 1 hr at 4 °C. NG2^+^ labeled cells were then FACS-purified as previously described (Influx, Cytopeia)^7^ and cultured under the same conditions as CNP-EGFP^+^ cells.

### Cell transfection with siRNA

Cell transfections were performed using the NeuroPORTER Transfection reagent (Genlantis, T400750) according to the manufacturer’s instructions. After WM dissection and dissociation, cells were plated in 12-well plates at a density of 50 cells/µl for 24 h. At the time of transfection, cell cultures were ∼60% confluent. Commercially available siRNA directed against Sirt1 (Ambion, NM-009870) and Sirt2 (Dharmacon, L-061727-02) used at 20pM produced selective gene knockdown at 7 h post-transfection. Briefly, 2 µl of 20pM siRNA solution and 12 µl of the transfection reagent were incubated in 100 µl of OptiMEM medium (Gibco) for 20 min in order to facilitate complex formation. The siRNA transfection mix was added to the cells cultured in 10% FBS. Controls were incubated with a non-specific siRNA (Silencer Negative control No 1, AM4635, Ambion). Cells were transfected for 7 h at 37 °C, washed with Hanks’ buffer and cultured in MEM with 10% FBS for an additional 24 h. The medium was then changed to Stem Cell Medium containing 20 ng/ml EGF and 10 ng/ml FGF. To demonstrate Sirt2 knockdown, Western blot analysis was performed on transfected Nx and Hx cells using anti-Sirt2 antibody. To assess the differentiation potential of cells, they were stained with anti-Olig2 and GalC antibodies and visualized by Rhodamine or TRITC-conjugated AffiniPure Goat Anti-Rabbit. Percentages of Olig2^+^ and GalC^+^ cells were quantified in random fields captured under 10x magnification (total of >250 cells) from at least three different samples and subjected to statistical analysis.

### Western blot and immunoprecipitation

Dissected WM samples from Nx and Hx mice were homogenized in RIPA lysis buffer with proteinase inhibitors (Santa Cruz Biotech, Inc, Sc 24948). Protein extracts were boiled for 5 min prior to loading onto 4–20% gradient gels (GeneMate, E4326-420, 20 µg of protein per each lane). Gels were electrotransfered to a 0.2 µm nitrocellulose membrane (Millipore). Blots were blocked in 5% milk in TBST for 1 h, and then incubated at 4 °C overnight with one of the following antibodies: anti-Cdk4 (1:5000, Santa Cruz, sc-260), -CyclinD1 (1:5000, Santa Cruz, sc25765), -p107 (1:2000, Santa Cruz, sc-65221), -E2F4 (1:2000, Santa Cruz, sc-866), -p27^Kip1^ (1:1000, Santa Cruz, sc-528), -Sirt1 (1:1000, Santa Cruz, sc-15404), -acetyl lysine (1:1000, Cell Signaling, 9681), -FoxO1 (1:5000, Cell Signaling, L27), -Actin (1:10000, Chemicon, MAB 1501 R), -Sirt2 (1:5000, Abcam, ab211033), -Sirt2 phospho Ser331 (1:5000, Active Motif, 61363), -p35 (1:5000, Abcam, ab123048), -Cdk5 (1:5000, Invitrogen, PAS-28795), and -p21^Cip1^ (1:1000, CalBiochem, OP76). Bands were detected with appropriate horseradish peroxide (HRP)-conjugated secondary antibodies, reacted with chemiluminescent ECL substrate (Amersham, RPN2132), and visualized by exposure to X-ray sensitive film. Band intensity was measured using the ImageJ program (NIH).

For ChIP antibody validation, five antibodies were taken under consideration including Abcam anti-Sirt2 antibodies #21033, #223534, #51023, and #67299, as wells as Sigma anti-Sirt2 antibody #S8447 (all 1:5000).

For immunoprecipitation, WM tissue extracts were prepared from Nx and Hx mice at P18 in RIPA buffer containing 2%Triton-X-100 and 0.2% SDS. Aliquots (270 µg tissue) were incubated overnight with antibodies against various antigens and 15 µl of Agarose A (Santa Cruz Biotechnology, sc 2001). Complexes bound to agarose A were collected by centrifugation and washed twice in 500 µl of RIPA buffer containing proteinase inhibitors. Precipitated proteins were analyzed by immunoblotting with antibodies against expected co-precipitating proteins. Bands were detected by using HRP labeled polyclonal anti-mouse or anti-rabbit antibodies (BD Biosciences 554002, 554021) and developed with a chemiluminescent substrate (ECL, Amersham).

### Quantitative PCR

Total RNA was isolated from FACS-purified cells from Nx and Hx WM using the RNeasy lipid tissue mini kit (Qiagen). Synthesis of cDNA was carried out using the iScript Reverse Transcription Supermix for RT-qPCR (Biorad). qPCR was performed on a CXF96 real-time system (Biorad) in a 20 µl reaction mixture using SsoAdvanced Universal SYBR Green PCR master mix (Biorad). Cycle parameters were 10 s at 95 °C and 30 s at 60 °C. Data were normalized to GAPDH. Primer sequences are as follows:

NG2: forward primer 5′-CGTGATGGTGTCTTTCGATG-3′, reverse primer 5′-GAGTACATCATGCCGACTGC-3′; Sirt1: forward primer 5′-GGTTGACTTAGGTCTTGTCTG-3′, reverse primer 5′-CGTCCCTTGTAATGTTTCCC-3′; Sirt2: forward primer 5′-AGCAAGGCACCA CTAGCCACC-3′; reverse primer 5′-TGTTCCTCTTTCTCTTTG-3′ ^81^;, CNP: forward primer 5′-CCGGAGACATAGTGCCCGCA-3′, reverse primer 5′-AAAGCTGGTCCAGCCGTTCC-3′; Gapdh: forward primer 5′-CTTTGTCAAGCTCATTTCCTGG-3′, reverse primer 5′-TCTTGCTCAGTGTCCTTGC-3′.

Primer sequences for validation of ChIP-Seq results are as follows:

Peak 268-3: forward primer 5′-TGTGTCAGGGTGTTTGCTTCT; reverse primer 5′-GGAGATCCGATGCCCCTTTC; Peak 268-2: forward primer 5′-TTGCCATAATTCAAGTGACCCA; reverse primer 5′-AAATCTGGTTTTGAGAACAGGGA; Peak 308: forward primer 5′-TGGTATGTGGCGAGGGCTTA; reverse primer 5′-ACTTGCTCCCACCAAAGCTC-3′.

### ChIP-Seq

The subcortical WM (SCWM) of WT mice from P12-P15 (reared under Nx and Hx conditions) was dissected and snap frozen. Chromatin was sonicated and immunoprecipitation with anti-Sirt2 antibody (Santa Cruz, sc211033) or IgG negative control was performed using the High Sensitivity ChIP kit from Abcam (Abcam 185913). DNA samples were sent to the Penn State Hershey Genome Sciences and Bioinformatics Facility and Penn State College of Medicine. Libraries were prepared using the NebNext kit for input of <100 ng DNA, according to the manufacturer’s protocol. 50 base pair, paired-end reads were obtained from the NovaSeq 6000 System (Illumina). Reads were mapped to the mouse reference genome (Genome Reference Consortium Mouse Build 38—mm10) using bowtie2 (version 2.4.4)^82^, and mitochondrial chromosomes were removed using Samtools (version 1.7)^83^. Duplicates were marked with Picard (version 2.26.10) and removed with Samtools. Binding peaks were identified using Macs2 (version 2.2.7.1)^84^ using an adjusted *p* value <0.1 as threshold. Sirt2 ChIP-seq data were compared with the RNA-seq data previously published^39^.

### RNAscope

RNAscope in situ hybridization was performed as previously described^85^. Briefly, the RNAscope multiplex fluorescent reagent kit V2 (323100) by Advanced Cell Diagnostics was used to perform fluorescent labeling of different RNA molecules according to the kit manual, with the following changes: H_2_O_2_ pretreatment for 5 min at room temperature, followed by washes in distilled water and 100% ethanol for 3 min. No target antigen retrieval step was performed. Slides were then dried at room temperature. RNAscope probes used were: *Pdgfrα* (480661), *Olig2* (447091-C3), *Enpp6* (511021-C2), *Diaph2* (custom), *Vegfc* (492701-C2), and *Sirt1* (418341). For the analysis, the corpus callosum was imaged using a Leica SP8 confocal microscope. Cells with at least two fluorescent puncta were counted as positive for that probe.

### Statistical analysis

All animal experiments include at least three animals per genotype and treatment group. Exact *n* values for each result are stated in the Figure legends. Quantifications are plotted as mean ± S.E.M. Significance levels for comparison between groups were determined with unpaired two-tailed Student’s *t* test or one-way ANOVA (with Tukey’s adjustment for multiple comparisons) where appropriate, using GraphPad Prism version 9 or Excel version 16.16.4 (Microsoft). Exact *p* values and statistical tests for each result are stated in the figure legends. Percent of reduction was calculated in the following manner: first, volumetric density of labeled cells was calculated by dividing their total number within a given z-stack column by its volume. Then, averages of volumetric densities within each group (Nx and Hx) were taken to calculate percent of reduction according to formula ((Hx–Nx/Nx) × 100). All experiments were replicated on numerous animals, collected across many days. The primary cellular and molecular findings were successfully replicated 3–4 times in both in vivo mouse studies and in vitro cell culture assays.

### Reporting summary

Further information on research design is available in the Nature Research Reporting Summary linked to this article.

## Supplementary information

Supplementary Information

Reporting Summary

## Data Availability

ChIP-seq data generated from this study have been deposited in the NCBI Sequence Read Archive (SRA) database with the accession code PRJNA773120. Source data are provided with this paper.

## Source data

Source Data File
