# Supplementary material for: Sirt2 promotes white matter oligodendrogenesis during development and in models of neonatal hypoxia
Source: Nat Commun. 2022 Aug 15;13:4771. doi: 10.1038/s41467-022-32462-2 (PMC9378658; doi:10.1038/s41467-022-32462-2)
Supplement: Supplementary file 1 — Supplementary Information [file 41467_2022_32462_MOESM1_ESM.pdf]

Sirt2 promotes white matter oligodendrogenesis during development and in models of neonatal hypoxia

Jablonska and Adams et al. *Nature Communications*

Supplementary Figures and Tables

Supplementary Figure 1

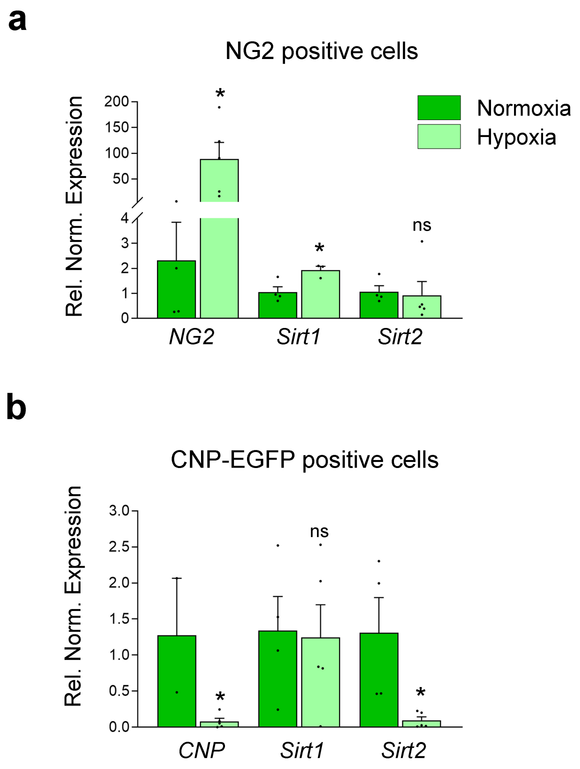

Supp. Figure 1. Sirt1 and Sirt2 expression levels in purified OPCs and OLs.

(a,b) Quantification of qPCR analysis in sorted NG2-expressing OPCs (NG2 *p=0.048, Sirt1 *p=0.026, Sirt2 ns p=0.84, n=4 Nx brains and 5 Hx brains, Student's t-test) **(a)** and CNP-EGFP$^+$ OLs (CNP *p=0.037, Sirt1 ns p=0.27, Sirt2 *p=0.027, n=4 Nx brains and 5 Hx brains, Student's t-test) **(b)** from Nx and Hx. Graphs display mean ± SEM values. Ns – not significant. All statistical tests are two-sided. Source data are provided as a Source Data file.

Supplementary Figure 2

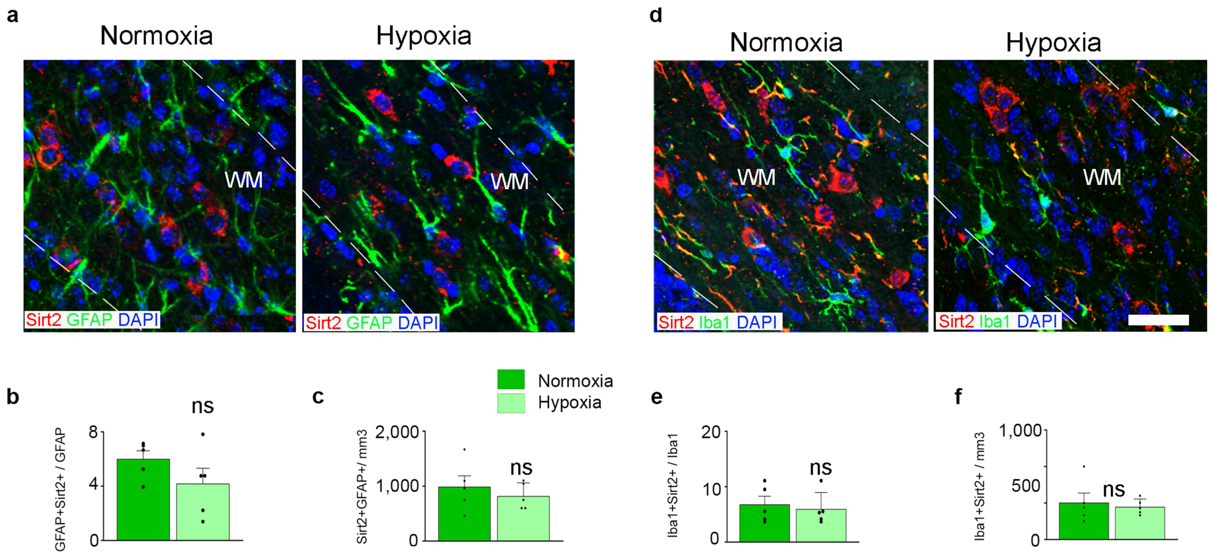

Supp. Figure 2. Hx does not affect Sirt2 expression in astrocytes or microglia.

(a,d) Coronal sections of subcortical WM in WT mice at P18 following Nx or Hx. Cells were immunolabeled with anti-GFAP **(a)** and anti-Iba **(d)** antibodies. Dotted lines delineate WM. WM, white matter. Scale bar=100µm. **(b,c)** Quantification of the percentage of $GFAP^{+}$ astrocytes expressing Sirt2 at P18 (ns p=0.1967, Student's t-test) **(b)** and their cell density (ns p=0.4821, Student's t-test) **(c)**. **(e,f)** Quantification of the percentage of $Iba1^{+}$ microglia expressing Sirt2 at P18 (ns p=0.6858, Student's t-test) **(e)** and their cell density (ns p=0.6970, Student's t-test) **(f)**. Graphs display mean ± SEM values (n=5 brains per condition). ns – not significant. All statistical tests are two-sided. Source data are provided as a Source Data file.

Supplementary Figure 3

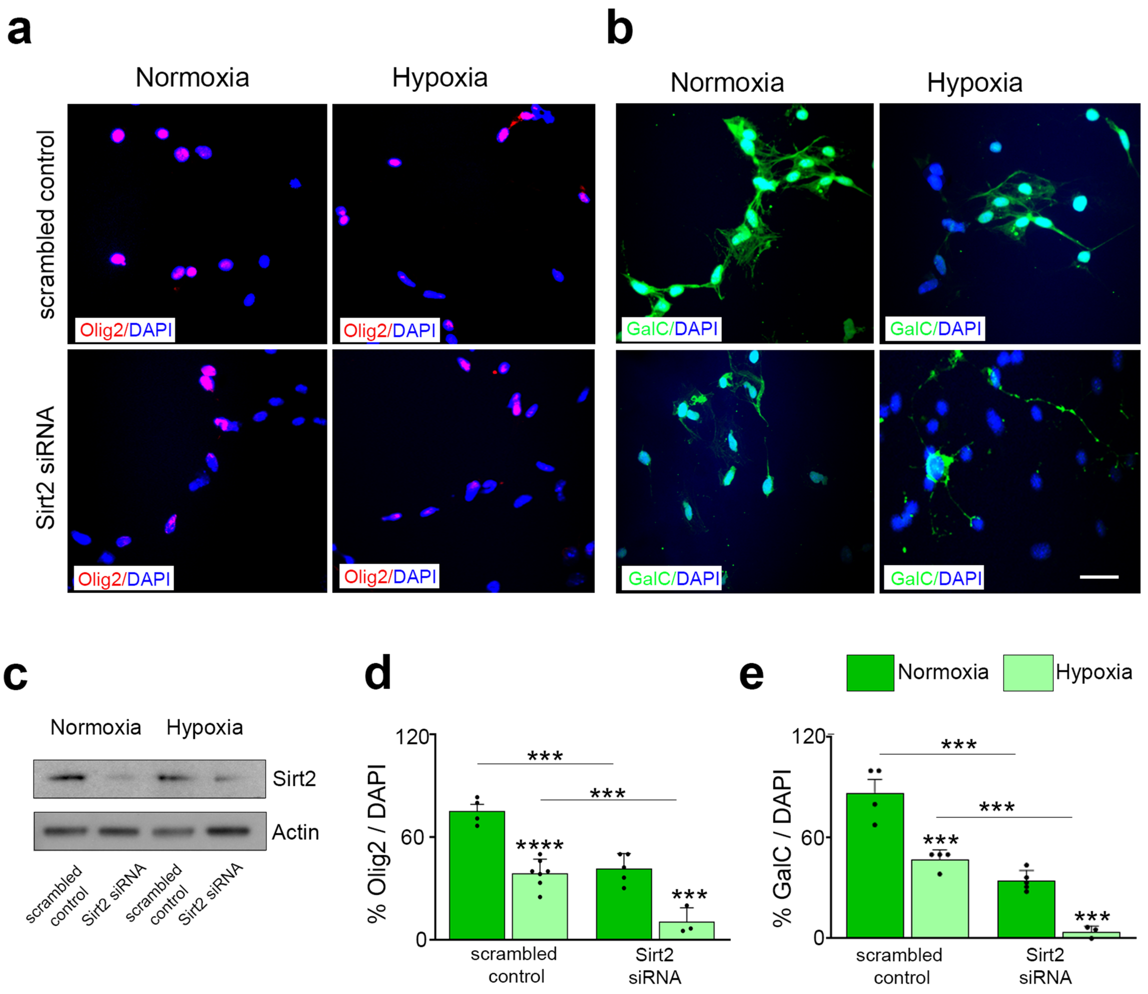

Supp. Figure 3. Sirt2 knockdown reduces OL differentiation in cultured WM cells.

(a,b) Nx and Hx WM cells were transfected with a Sirt2 siRNA or a scrambled siRNA control and analyzed for expression of Olig2 **(a)** and GalC **(b)**. **(c)** Western blot for Sirt2 protein in transfected WM cells, confirming Sirt2 knockdown (n=1 experiment, 3 mice per condition). **(d)** Quantification of the percentage of Olig2^{+} WM cells following transfection of scrambled control and Sirt2 siRNA (Nx vs Hx control ****p<0.0001, Nx vs Hx siRNA ***p=0.0006, Nx control vs Nx siRNA ***p<0.0001, Hx control vs Hx siRNA ***p=0.0008, ANOVA with Tukey's multiple comparisons adjustment). **(e)** Quantification of the percentage of GalC^{+} WM cells following transfection of scrambled control and Sirt2 siRNA (Nx vs Hx control ***p=0.0003, Nx vs Hx siRNA ***p=0.0001, Nx control vs Nx siRNA ***p<0.0004, Hx control vs Hx siRNA ***p=0.0003, ANOVA with Tukey's multiple comparisons adjustment). Graphs display mean ± SEM values (n=3 mice per condition). All statistical tests are two-sided. Source data are provided as a Source Data file.

Supplementary Figure 4

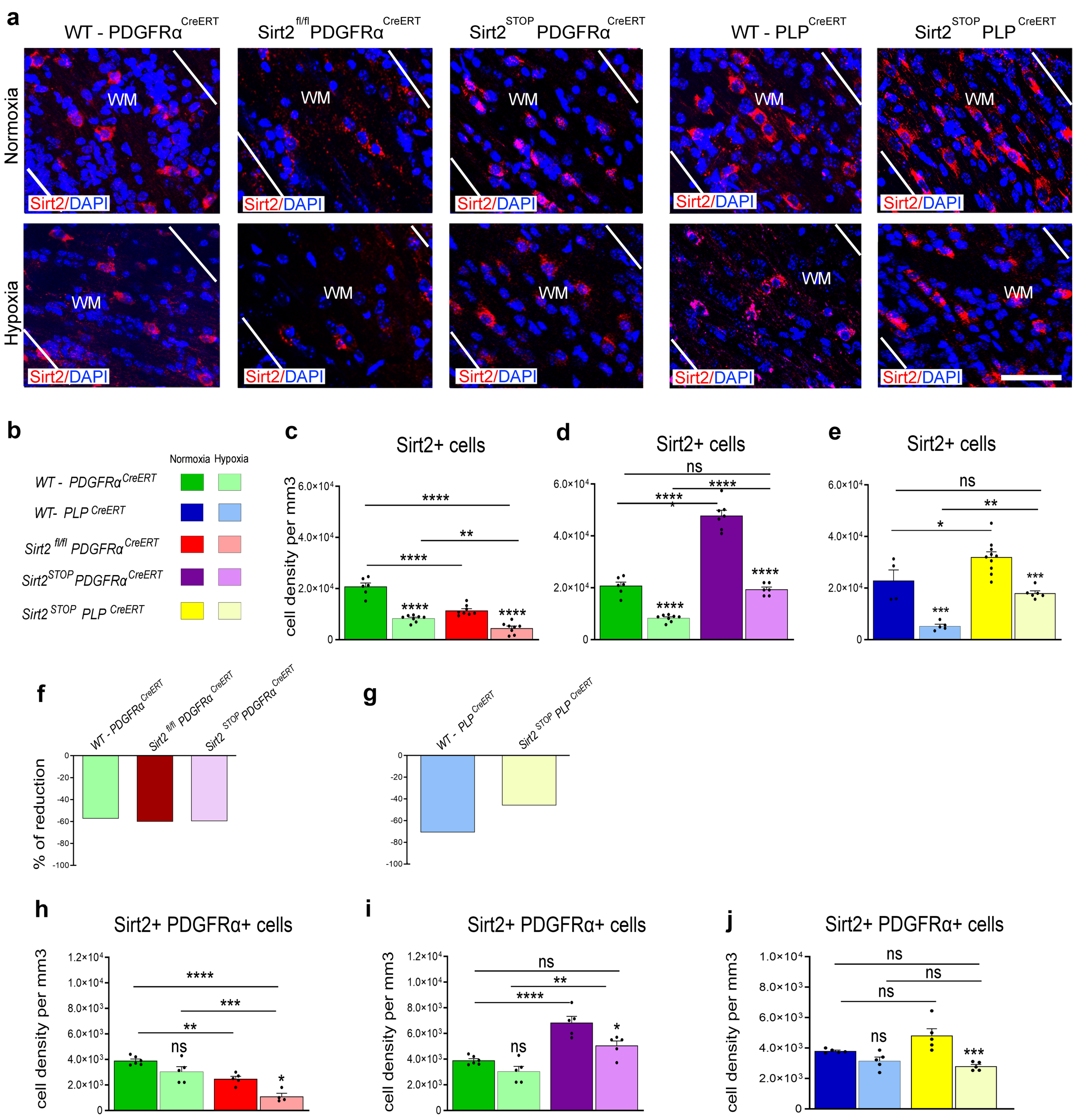

Supp. Figure 4. Modulating Sirt2 expression levels in the subcortical WM.

(a) Coronal sections of subcortical WM from Sirt2$^{fl/fl}$PDGFRα^{CreERT}, Sirt2^{STOP}PDGFRα^{CreERT}, Sirt2^{STOP}PLP^{CreERT}, and their respective controls at P18 following Nx or Hx. White lines delineate WM, WM-white matter. Scale bar=100μm. **(b)** Color legend for different transgenic mice and their controls in Nx and Hx conditions. **(c)** Quantification of the cell density of Sirt2^{+} WM cells in WT and Sirt2$^{fl/fl}$PDGFRα^{CreERT} mice (WT: Nx vs Hx ****p<0.0001, Sirt2$^{fl/fl}$PDGFRα^{CreERT}: Nx vs Hx ****p<0001, WT Nx vs Sirt2$^{fl/fl}$PDGFRα^{CreERT} Nx ****p<0.0001, WT Hx vs Sirt2$^{fl/fl}$PDGFRα^{CreERT} Hx *p=0.0139, WT Nx vs Sirt2$^{fl/fl}$PDGFRα^{CreERT} Hx ****p<0001, n=6 WT-Nx, 8 WT-Hx, 8 Sirt2$^{fl/fl}$-Nx, 8Sirt2$^{fl/fl}$-Hx mice,

ANOVA with Tukey's multiple comparisons adjustment). **(d)** Quantification of the cell density of $Sirt2^+$ WM cells in WT and $Sirt2^{STOP}PDGFR\alpha^{CreERT}$ mice (WT: Nx vs Hx ****p<0.0001, $Sirt2^{STOP}PDGFR\alpha^{CreERT}$: Nx vs Hx ****p<0001, WT Nx vs $Sirt2^{STOP}PDGFR\alpha^{CreERT}$ Nx ****p<0.0001, WT Hx vs $Sirt2^{STOP}PDGFR\alpha^{CreERT}$ Hx ****p<0.0001, WT Nx vs $Sirt2^{STOP}PDGFR\alpha^{CreERT}$ Hx ns p=0.8981, n=7 WT-Nx, 8 WT-Hx, 7 $Sirt2^{STOP}$-Nx, 6 $Sirt2^{STOP}$-Hx mice, ANOVA with Tukey's multiple comparisons adjustment). **(e)** Quantification of the cell density of $Sirt2^+$ WM cells in WT and $Sirt2^{STOP}PLP^{CreERT}$ mice (WT: Nx vs Hx ***p<0.0004, $Sirt2^{STOP}PLP^{CreERT}$: Nx vs Hx ***p=0.0003, WT Nx vs $Sirt2^{STOP}PLP^{CreERT}$ Nx *p<0.0407, WT Hx vs $Sirt2^{STOP}PLP^{CreERT}$ Hx **p<0.0039, WT Nx vs $Sirt2^{STOP}PLP^{CreERT}$ Hx ns p=0.5164, n=6 WT-Nx, 6 WT-Hx, 6 $Sirt2^{STOP}$-Nx, 5 $Sirt2^{STOP}$-Hx mice, ANOVA with Tukey's multiple comparisons adjustment). **(f,g)** Quantification of the percentage of reduction of $Sirt2^+$ cells in each transgenic mouse strain following Hx. **(h)** Quantification of the cell density of $Sirt2^+PDGFR\alpha^+$ WM cells in WT and $Sirt2^{fl/fl}PDGFR\alpha^{CreERT}$ mice (WT: Nx vs Hx ns p=0.0982, $Sirt2^{fl/fl}PDGFR\alpha^{CreERT}$: Nx vs Hx *p=0.0115, WT Nx vs $Sirt2^{fl/fl}PDGFR\alpha^{CreERT}$ Nx **p=0.0038, WT Hx vs $Sirt2^{fl/fl}PDGFR\alpha^{CreERT}$ Hx *** p=0.006, WT Nx vs $Sirt2^{fl/fl}PDGFR\alpha^{CreERT}$ Hx ****p<0001, n=6 WT-Nx, 5 WT-Hx, 5 $Sirt2^{fl/fl}$-Nx, 4 $Sirt2^{fl/fl}$-Hx mice, ANOVA with Tukey's multiple comparisons adjustment). **(i)** Quantification of the cell density of $Sirt2^+PDGFR\alpha^+$ WM cells in WT and $Sirt2^{STOP}PDGFR\alpha^{CreERT}$ mice (WT: Nx vs Hx ns p=0.3245, $Sirt2^{STOP}PDGFR\alpha^{CreERT}$: Nx vs Hx *p=0.0124, WT Nx vs $Sirt2^{STOP}PDGFR\alpha^{CreERT}$ Nx ****p<0.0001, WT Hx vs $Sirt2^{STOP}PDGFR\alpha^{CreERT}$ Hx **p=0.0052, WT Nx vs $Sirt2^{STOP}PDGFR\alpha^{CreERT}$ Hx ns p=0.1229, n=6 WT-Nx, 5 WT-Hx, 5 $Sirt2^{STOP}$-Nx, 5 $Sirt2^{STOP}$-Hx mice, ANOVA with Tukey's multiple comparisons adjustment). **(j)** Quantification of the cell density of $Sirt2^+PDGFR\alpha^+$ WM cells in WT and $Sirt2^{STOP}PLP^{CreERT}$ mice (WT: Nx vs Hx ns p=0.3495, $Sirt2^{STOP}PLP^{CreERT}$: Nx vs Hx ***p=0.0003, WT Nx vs $Sirt2^{STOP}PLP^{CreERT}$ Nx ns p=0.0660, WT Hx vs $Sirt2^{STOP}PLP^{CreERT}$ Hx ns p=0.7709, WT Nx vs $Sirt2^{STOP}PLP^{CreERT}$ Hx ns p=0.0698, n=5 mice per group, ANOVA with Tukey's multiple comparisons adjustment). Graphs display mean ± SEM values, except for percent reduction graphs. All statistical tests are two-sided. Source data are provided as a Source Data file.

Supplementary Figure 5

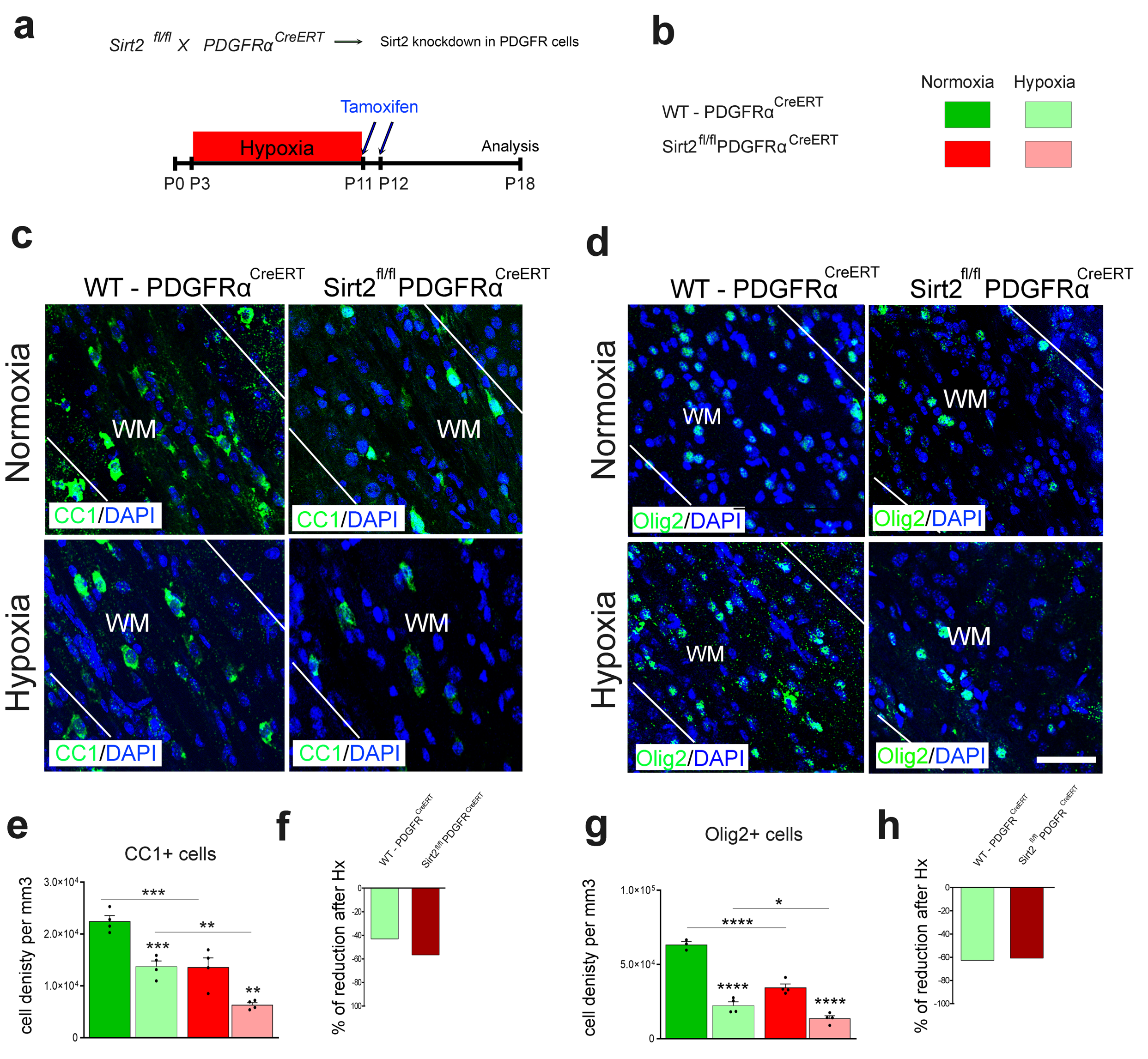

Supp. Figure 5. Sirt2 ablation reduces oligodendrogenesis in vivo.

(a) Experimental schematic for Sirt2 knockdown in PDGFRα^{+} OPCs. **(b)** Color legend for transgenic mice in Nx and Hx conditions. **(c,d)** Coronal sections of subcortical WM from Sirt2$^{fl/fl}$PDGFRα^{CreERT} and WT mice at P18 following Nx and Hx. White lines delineate WM, WM-white matter. Scale bar=100µm. **(e)** Quantification of the cell density of CC1^{+} cells in WM at P18 (WT: Nx vs Hx ***p=0.0009, Sirt2$^{fl/fl}$PDGFRα^{CreERT}: Nx vs Hx **p=0.0052, WT Nx vs Sirt2$^{fl/fl}$PDGFRα^{CreERT} Nx ***p=0.0008, WT Hx vs Sirt2$^{fl/fl}$PDGFRα^{CreERT} Hx **p=0.0042, n=4 mice per group, ANOVA with Tukey's multiple comparisons adjustment). **(g)** Quantification of the cell density of Olig2^{+} cells in WM at P18 (WT: Nx vs Hx ****p<0.0001, Sirt2$^{fl/fl}$PDGFRα^{CreERT}: Nx vs Hx ****p<0.0001, WT Nx vs Sirt2$^{fl/fl}$PDGFRα^{CreERT} Nx ****p<0.0001, WT Hx vs Sirt2$^{fl/fl}$PDGFRα^{CreERT} Hx *p<0.0255, n=3 WT-Nx, 4 WT-Hx, 4 Sirt2$^{fl/fl}$-Nx, 4 Sirt2$^{fl/fl}$-Hx mice, ANOVA with Tukey's multiple comparisons adjustment). **(f,h)** Quantification of the

percentage of reduction of CC1^{+} cells **(f)** and Olig2^{+} cells **(h)** after Hx. Graphs display mean ± SEM values, except for percent reduction graphs. All statistical tests are two-sided. Source data are provided as a Source Data file.

Supplementary Figure 6

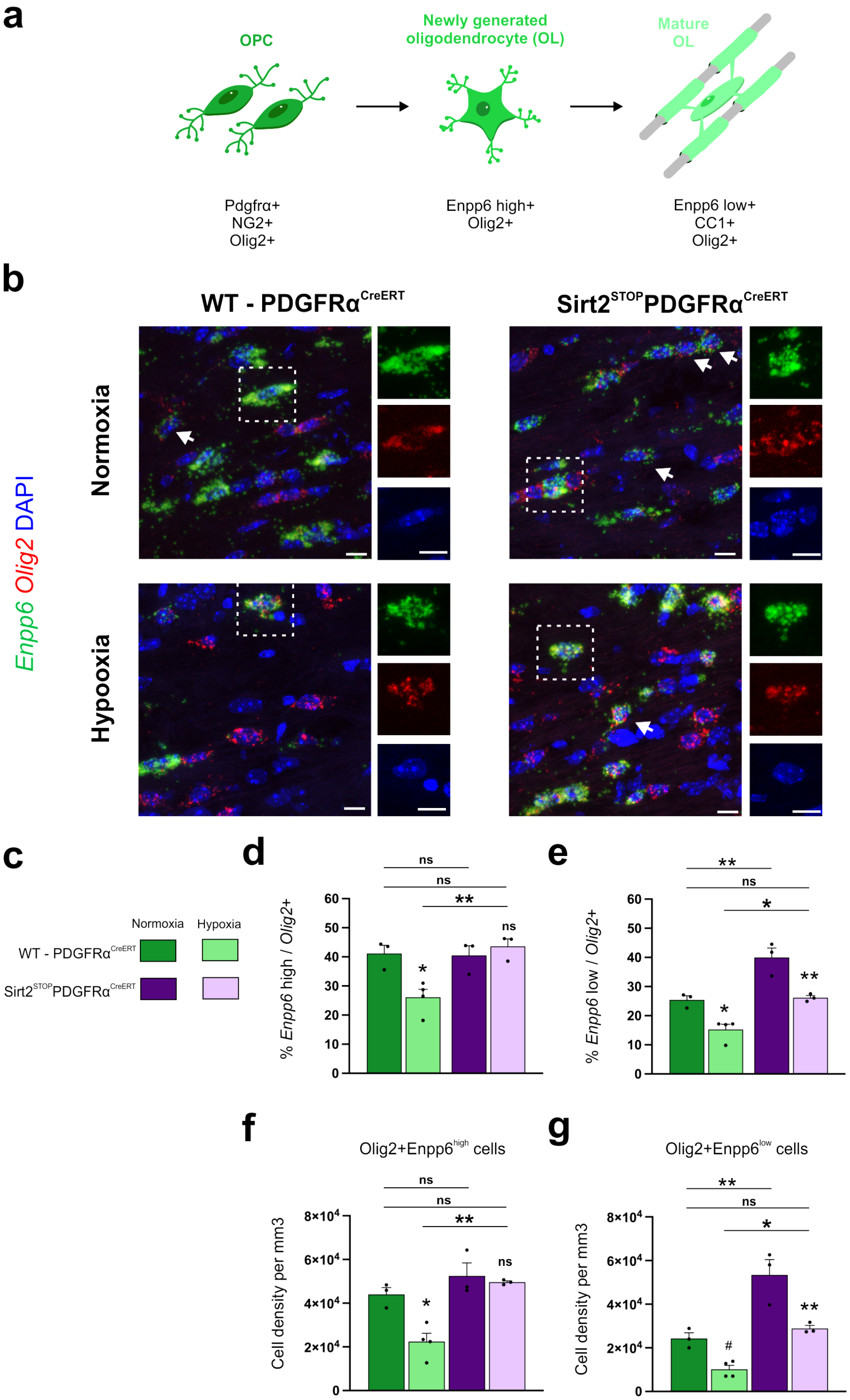

Supp. Figure 6. Sirt2 overexpression in OPCs rescues newly-generated OLs following Hx.

(a) Schematic illustrating the expression pattern of several commonly used markers for OL lineage progression. **(b)** Coronal sections of corpus callosum from WT and Sirt2^{STOP}PDGFRα^{CreERT} mice at P18

following Nx and Hx conditions. Boxes represent magnified single-channel images of examples of *Enpp6*$^{+}$ high-expressing OLs under each condition. Arrows point to *Enpp6*$^{+}$ low-expressing OLs. Scale bars=10um. **(c)** Color legend for transgenic mice in Nx and Hx conditions. **(d)** Quantification of the percentage of *Enpp6*$^{+}$ high-expressing newly generated OLs at P18 (WT: Nx vs Hx *p=0.018, Sirt2^{STOP}PDGFRα^{CreERT}: Nx vs Hx ns p=0.89, WT Nx vs Sirt2^{STOP}PDGFRα^{CreERT} Nx: ns p=0.99, WT Nx vs Sirt2^{STOP}PDGFRα^{CreERT} Hx: ns p=0.94, WT Hx vs Sirt2^{STOP}PDGFRα^{CreER} Hx: **p=0.0076, ANOVA with Tukey's multiple comparisons adjustment). **(e)** Quantification of the percentage of *Enpp6*$^{+}$ low-expressing mature OLs at P18 (WT: Nx vs Hx *p=0.023, Sirt2^{STOP}PDGFRα^{CreERT}: Nx vs Hx **p=0.0054, WT Nx vs Sirt2^{STOP}PDGFRα^{CreERT} Nx: **p=0.0038, WT Nx vs Sirt2^{STOP}PDGFRα^{CreERT} Hx: ns p=0.99, WT Hx vs Sirt2^{STOP}PDGFRα^{CreER} Hx: *p=0.015, ANOVA with Tukey's multiple comparisons adjustment). **(f)** Quantification of the total number of *Olig2*$^{+}$*Enpp6*$^{+}$ high-expressing newly generated OLs at P18 (WT: Nx vs Hx *p=0.013, Sirt2^{STOP}PDGFRα^{CreERT}: Nx vs Hx ns p=0.96, WT Nx vs Sirt2^{STOP}PDGFRα^{CreERT} Nx: ns p=0.49, WT Nx vs Sirt2^{STOP}PDGFRα^{CreERT} Hx: ns p=0.77, WT Hx vs Sirt2^{STOP}PDGFRα^{CreER} Hx: **p=0.0031, ANOVA with Tukey's multiple comparisons adjustment). **(g)** Quantification of the total number of *Olig2*$^{+}$*Enpp6*+ low-expressing mature OLs at P18 (WT: Nx vs Hx $^{\#}$p=0.078, Sirt2^{STOP}PDGFRα^{CreERT}: Nx vs Hx **p=0.0059, WT Nx vs Sirt2^{STOP}PDGFRα^{CreERT} Nx: **p=0.0019, WT Nx vs Sirt2^{STOP}PDGFRα^{CreERT} Hx: ns p=0.83, WT Hx vs Sirt2^{STOP}PDGFRα^{CreER} Hx: *p=0.0197, ANOVA with Tukey's multiple comparisons adjustment). Graphs display mean ± SEM values (n=3 animals for WT-Nx, Sirt2^{STOP}-Nx, and Sirt2^{STOP}-Hx groups, n=4 animals for WT-Hx group). All statistical tests are two-sided. Source data are provided as a Source Data file. The schematic in (a) was created using CorelDraw 2018 software (version 20.1.0.708).

Supplementary Figure 7

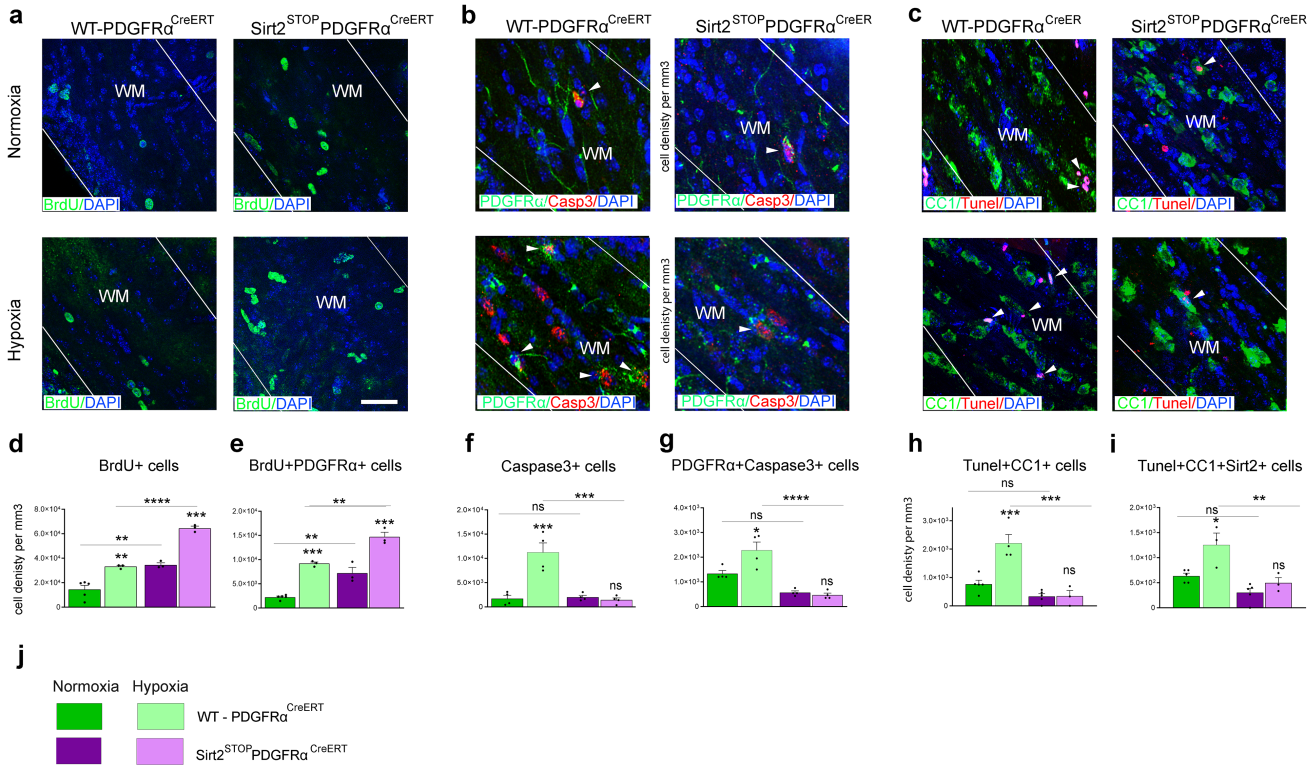

Supp. Figure 7. Sirt2 overexpression in OPCs increases proliferation and reduces apoptosis. **(a-c)** Coronal sections of subcortical WM from Sirt2^{STOP}PDGFRα^{CreERT} and WT mice at P18 stained with anti-BrdU antibody **(a)**, anti-PDGFRα and -Caspase3 antibodies **(b)**, and anti-CC1 antibody and Tunel **(c)**. Dotted lines delineate WM, WM-white matter. Scale bar=100µm. **(d)** Quantification of the cell density of BrdU$^+$ cells in WM at P18 (WT: Nx vs Hx **p=0.0028, Sirt2^{STOP}PDGFRα^{CreERT}: Nx vs Hx ***p=0.0001, WT Nx vs Sirt2^{STOP}PDGFRα^{CreERT} Nx **p=0.0016, WT Hx vs Sirt2^{STOP}PDGFRα^{CreERT} Hx ****p<0.0001, ANOVA with Tukey's multiple comparisons adjustment). **(e)** Quantification of the cell density of BrdU$^+$PDGFRα$^+$ cells in WM at P18 (WT: Nx vs Hx ***p=0.0001, Sirt2^{STOP}PDGFRα^{CreERT}: Nx vs Hx ***p=0.0001, WT Nx vs Sirt2^{STOP}PDGFRα^{CreERT} Nx **p=0.0015, WT Hx vs Sirt2^{STOP}PDGFRα^{CreERT} Hx **p=0.0016, ANOVA with Tukey's multiple comparisons adjustment). **(f)** Quantification of the cell density of Caspase3$^+$ cells in WM at P18 (WT: Nx vs Hx ***p=0.0002, Sirt2^{STOP}PDGFRα^{CreERT}: Nx vs Hx ns p=0.9802, WT Nx vs Sirt2^{STOP}PDGFRα^{CreERT} Nx ns p=0.9973, WT Hx vs Sirt2^{STOP}PDGFRα^{CreERT} Hx ***p=0.0002, ANOVA with Tukey's multiple comparisons adjustment). **(g)** Quantification of the cell density of Caspase3$^+$PDGFRα$^+$ cells in WM at P18 (WT: Nx vs Hx *p=0.0164, Sirt2^{STOP}PDGFRα^{CreERT}: Nx vs Hx ns p=0.9787, WT Nx vs Sirt2^{STOP}PDGFRα^{CreERT} Nx ns p=0.0553, WT Hx vs Sirt2^{STOP}PDGFRα^{CreERT} Hx ****p<0.0001, ANOVA with Tukey's multiple comparisons adjustment). **(h)** Quantification of the cell density of Tunel$^+$CC1$^+$ cells in WM at P18 (WT: Nx vs Hx ***p=0.0005, Sirt2^{STOP}PDGFRα^{CreERT}: Nx vs Hx ns p>0.9999, WT Nx vs Sirt2^{STOP}PDGFRα^{CreERT} Nx ns p=0.3267, WT

Hx vs Sirt2^{STOP}PDGFRα^{CreERT} Hx ***p=0.0001, ANOVA with Tukey's multiple comparisons adjustment). **(i)** Quantification of the cell density of Tunel^{+}CC1^{+}Sirt2^{+} cells in WM at P18 (WT: Nx vs Hx *p=0.0124, Sirt2^{STOP}PDGFRα^{CreERT}: Nx vs Hx ns p=0.6534, WT Nx vs Sirt2^{STOP}PDGFRα^{CreERT} Nx ns p=0.1470, WT Hx vs Sirt2^{STOP}PDGFRα^{CreERT} Hx **p=0.0067, ANOVA with Tukey's multiple comparisons adjustment). Graphs display mean ± SEM values (n=3 brains per condition). **(j)** Color legend for transgenic mice and their littermates in Nx and Hx conditions. All statistical tests are two-sided. Source data are provided as a Source Data file.

Supplementary Figure 8

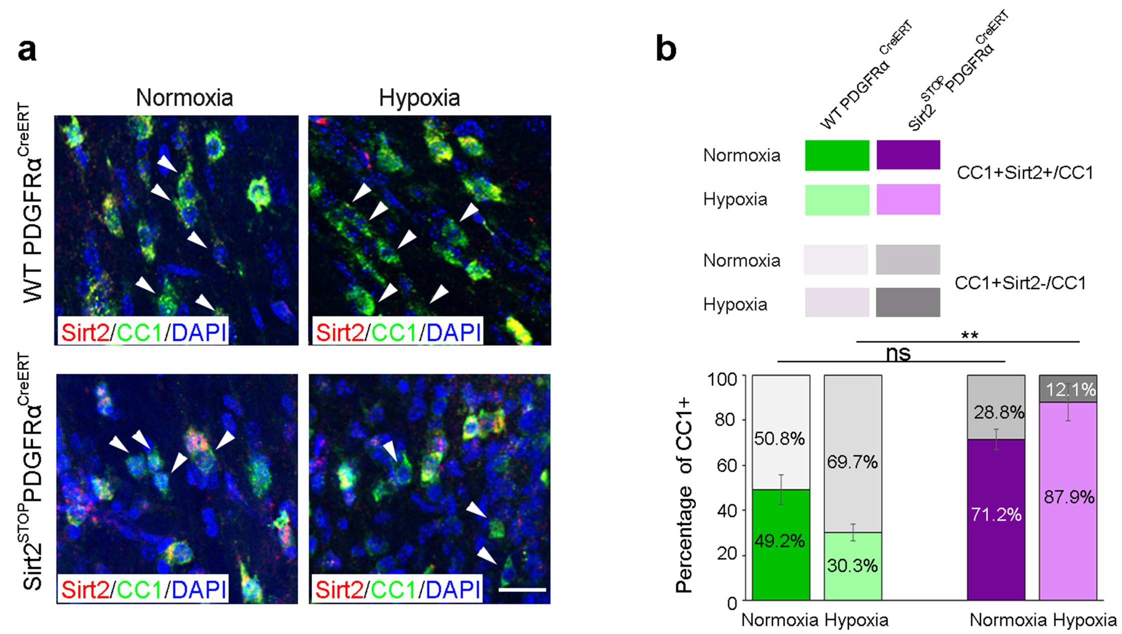

Supp. Figure 8. Distribution of Sirt2 expression in mature OLs.

(a) Coronal sections of subcortical WM from Sirt2^{STOP}PDGFRα^{CreERT} and WT mice at P18 after Nx and Hx. White arrows indicate CC1^{+} Sirt2^{-} cells**.** Scale bar=50µm. **(b)** Quantification of the percentage of CC1^{+} OLs that are Sirt2^{+} and Sirt2^{-} in Nx and Hx mice, in both Sirt2^{STOP}PDGFRα^{CreERT} and WT mice (CC1^{+}Sirt2^{-} cells: WT Nx vs Sirt2^{STOP}PDGFRα^{CreERT} Nx ns p=0.1848, WT Hx vs Sirt2^{STOP}PDGFRα^{CreERT} Hx **p=0.0015, ANOVA with Tukey's multiple comparisons adjustment). Graph displays mean ± SEM values (n=3 brains per condition). Statistical tests are two-sided. Source data are provided as a Source Data file.

Supplementary Figure 9

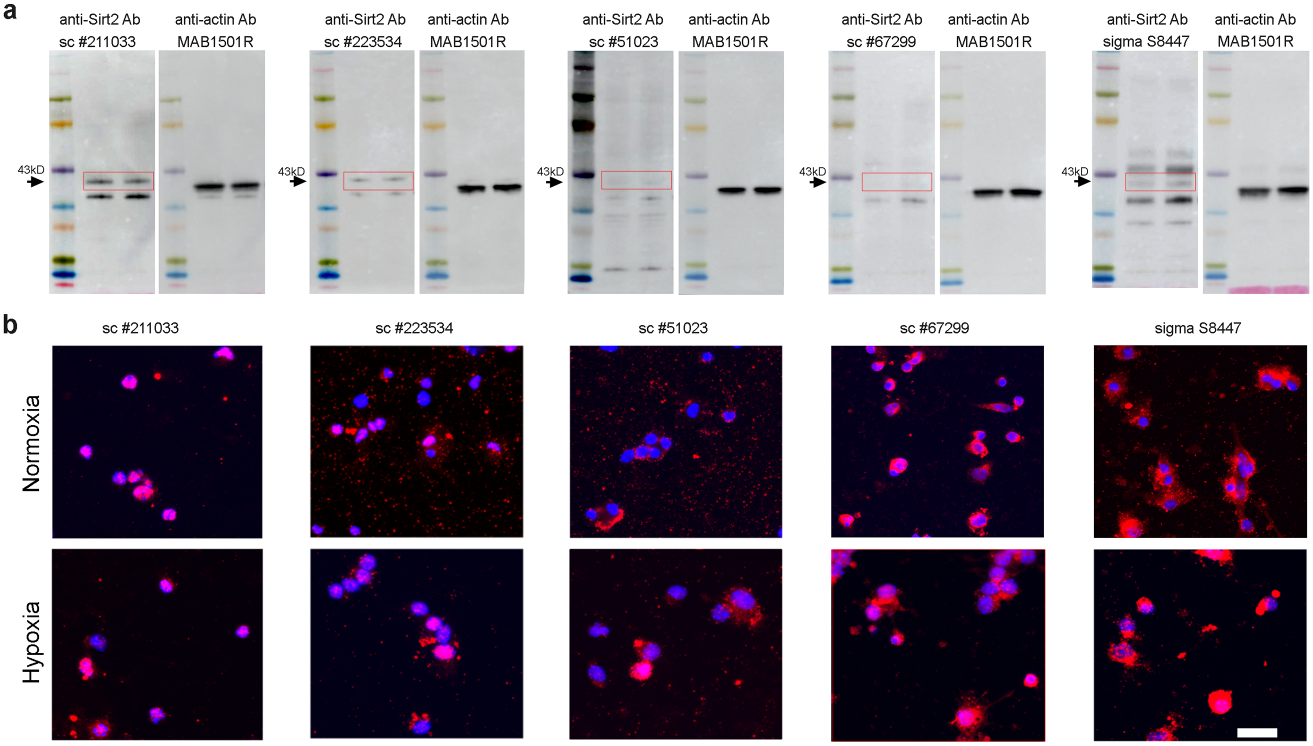

Supp. Figure 9. Validation of anti-Sirt2 antibodies for ChIP-seq.

(a) Five anti-Sirt2 antibodies were analyzed by Western blot for their ability to detect Sirt2 protein from dissected subcortical WM. The red box outlines the correct band (43KD). (n=1 experiment per antibody, 3 mice per condition). **(b)** Immunocytochemistry for Sirt2 performed on cultured cells from Nx and Hx WM cells. Scale bar=50µm. The most specific antibody was anti-Sirt2 antibody (# ab211033), which produced two distinct bands on Western blot and nuclear staining of Hx WM cells. Source data are provided as a Source Data file.

Supplementary Figure 10

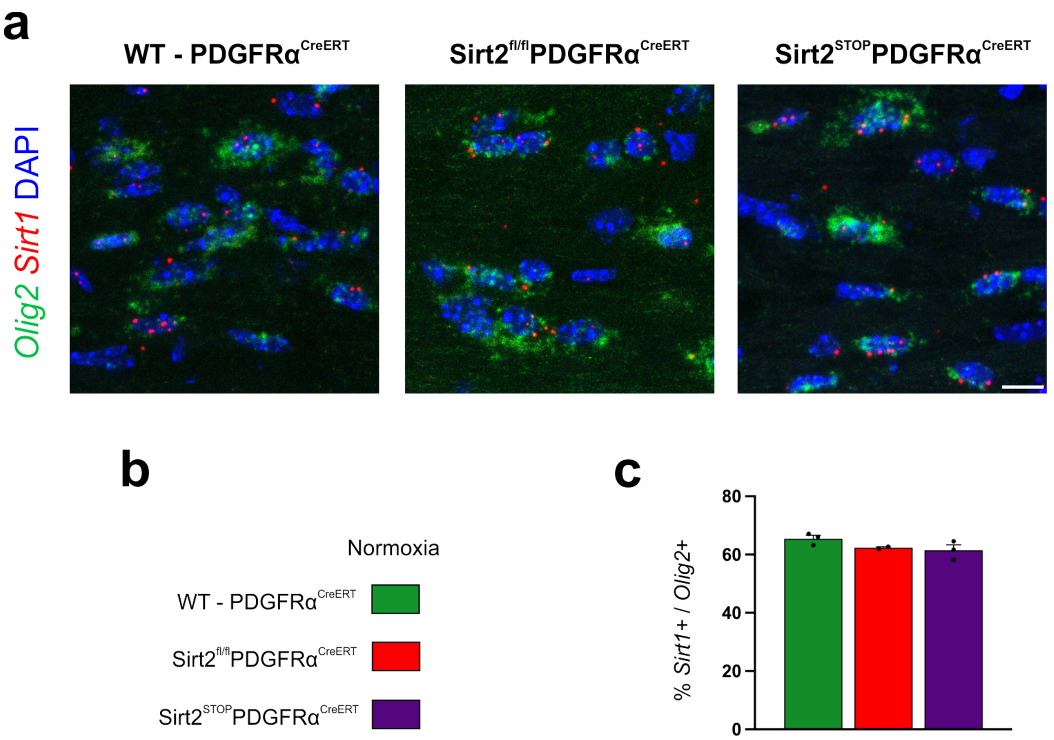

Supp. Figure 10. Sirt1 expression in Sirt2 knockout and overexpression mice.

(a) RNAscope images of *Sirt1* mRNA expression (red puncta) in *Olig2*$^+$ OL lineage cells of WT, Sirt2$^{fl/fl}$PDGFRα^{CreERT}, and Sirt2^{STOP}PDGFRα^{CreERT} mice at P18 under Nx conditions. Scale bar=10um. **(b)** Color legend for transgenic mice. **(c)** Quantification of the percentage of *Sirt1*$^+$ WM OL lineage cells at P18 (n=3 WT, 2 Sirt2floxed, 3 Sirt2^{STOP} mice). Graph displays mean ± SEM. Source data are provided as a Source Data file.

Supplementary Table 1

Gender	Gestational Age (weeks)	Cause of death
F	40	Congenital cardiac disease
F	40	Congenital cardiac disease
M	40	Congenital diaphragmatic hernia
F	40	Congenital cardiac disease
F	27	Acute respiratory failure
F	32	Acute respiratory failure
M	24	Pulmonary hypertension
F	24	Acute respiratory failure

Supp. Table 1. Human tissue samples.

Brain tissue samples were analyzed from four term (all gestational age of 40 weeks) and four preterm (gestational ages 24-32 weeks) human neonates for H&E and immunohistochemistry analysis in Figure 2. F, female. M, male.

Supplementary Table 2

Comparison	Peak ID	Chromosome	Start	End	Nearest promoter (gene name)
Nx versus IgG	255	chr9	3006836	3007092	Mir101c
Nx versus IgG	307	chr9	3024173	3024479	Mir101c
Hx versus IgG	230	chrX	148573087	148573315	Gm15114
Hx versus IgG	256	chrX	146849363	146849617	Htr2c
Hx versus IgG	228	chr1	143841419	143841645	Uchl5
Hx versus IgG	352	chrX	129441381	129441731	Diaph2
Hx versus IgG	222	chr14	123413630	123413850	Nalcn
Hx versus IgG	222-2	chr7	114941400	114941620	A730082K24Rik
Hx versus IgG	363	chr3	111841231	111841592	Gm6602
Hx versus IgG	180	chr14	105995885	105996063	Spry2
Hx versus IgG	384	chr6	105904769	105905151	Cntn4
Hx versus IgG	253	chr15	103632669	103632920	Glycam1
Hx versus IgG	226	chr1	102642361	102642585	Gm20268
Hx versus IgG	304	chr6	101452853	101453155	Pdzrn3
Hx versus IgG	308	chr3	100134500	100134806	Wdr3
Hx versus IgG	277	chr2	98666944	98667219	Lrrc4c
Hx versus IgG	426	chr2	98662331	98662755	Lrrc4c
Hx versus IgG	184-2	chr2	96869531	96869713	Lrrc4c
Hx versus IgG	202	chr4	92497176	92497376	Izumo3
Hx versus IgG	201	chr4	91736323	91736522	Elavl2
Hx versus IgG	328	chrX	90001161	90001487	4930415L06Rik
Hx versus IgG	241	chr14	89846797	89847036	Gm5088
Hx versus IgG	307-2	chr13	86322736	86323041	Cox7c
Hx versus IgG	349	chrX	82190270	82190617	4930595M18Rik
Hx versus IgG	239	chr9	79262653	79262890	Gm10635
Hx versus IgG	300	chr17	76213895	76214193	Gm4710
Hx versus IgG	376	chr3	73328071	73328445	4930509J09Rik
Hx versus IgG	268	chr15	71110634	71110900	Fam135b
Hx versus IgG	301	chrX	70721262	70721561	Gm14725
Hx versus IgG	245	chr4	68226344	68226587	Gm12911
Hx versus IgG	205	chr8	65730647	65730850	March1
Hx versus IgG	438	chr16	63459050	63459486	Epha3
Hx versus IgG	279	chr13	59403887	59404164	Agtpbp1
Hx versus IgG	188	chr19	58833088	58833274	Hspa12a
Hx versus IgG	456	chr12	58294507	58294961	Clec14a
Hx versus IgG	263	chr10	56175530	56175791	Tbc1d32
Hx versus IgG	254	chrX	50996712	50996964	Rap2c
Hx versus IgG	307	chr10	50932280	50932585	Sim1
Hx versus IgG	184	chr18	47827628	47827810	Gm4146
Hx versus IgG	243	chr3	47451434	47451675	Mir6379
Hx versus IgG	264	chr10	41640410	41640672	5730435O14Rik
Hx versus IgG	231	chr1	41330805	41331034	
Hx versus IgG	295	chrX	35649644	35649937	Dock11
Hx versus IgG	270	chr9	30638775	30639043	Snx19
Hx versus IgG	238	chr6	26121952	26122188	Gm20756

Comparison	Peak ID	Chromosome	Start	End	Nearest promoter (gene name)
Hx versus IgG	251	chr18	17558426	17558675	4930545E07Rik
Hx versus IgG	175	chr13	17473675	17473848	Sugct
Hx versus IgG	207	chr7	15654173	15654378	Obox3
Hx versus IgG	357	chr9	3024133	3024488	Mir101c
Hx versus IgG	274	chr9	3020262	3020534	Mir101c
Hx versus IgG	284	chr9	3006824	3007106	Mir101c
Hx versus IgG	786	chr9	3000545	3001329	Mir101c
Hx versus IgG	248	chrJH584304.1	90261	90507	
Hx versus IgG	305	chrJH584304.1	87867	88170	
Hx versus Nx	244	chrX	148573073	148573315	Gm15114
Hx versus Nx	268-3	chrX	129441387	129441653	Diaph2
Hx versus Nx	280	chr14	123413579	123413857	Nalcn
Hx versus Nx	385	chr3	111841237	111841620	Gm6602
Hx versus Nx	251	chr6	105904860	105905109	Cntn4
Hx versus Nx	215	chr1	102642341	102642554	Gm20268
Hx versus Nx	304	chr6	101452853	101453155	Pdzrn3
Hx versus Nx	375	chr2	98666944	98667317	Lrrc4c
Hx versus Nx	600	chr2	98662265	98662863	Lrrc4c
Hx versus Nx	268-2	chr4	91736250	91736516	Elavl2
Hx versus Nx	349	chrX	82190270	82190617	4930595M18Rik
Hx versus Nx	228	chr9	79262655	79262881	Gm10635
Hx versus Nx	217	chr17	76213916	76214131	Gm4710
Hx versus Nx	173	chr3	73328189	73328360	4930509J09Rik
Hx versus Nx	253	chr8	72988053	72988304	Mir28b
Hx versus Nx	256	chr15	71110646	71110900	Fam135b
Hx versus Nx	208	chrX	70721262	70721468	Gm14725
Hx versus Nx	195-2	chr5	69882594	69882787	Gnpda2
Hx versus Nx	246	chr8	65730647	65730891	March1
Hx versus Nx	195	chr19	58833081	58833274	Hspa12a
Hx versus Nx	268	chr12	58294568	58294834	Clec14a
Hx versus Nx	231	chr15	56589841	56590070	Has2os
Hx versus Nx	176	chr8	53901846	53902020	Vegfc
Hx versus Nx	308	chrX	50996694	50997000	Rap2c
Hx versus Nx	235	chr10	50932277	50932510	Sim1
Hx versus Nx	199	chr1	50292133	50292330	
Hx versus Nx	295	chrX	35649644	35649937	Dock11
Hx versus Nx	210	chr18	17558464	17558672	4930545E07Rik
Hx versus Nx	198	chr8	8287944	8288140	Efnb2
Hx versus Nx	400	chr9	3032457	3032855	Mir101c
Hx versus Nx	407	chr9	3024114	3024519	Mir101c
Hx versus Nx	276	chr9	3020262	3020536	Mir101c
Hx versus Nx	413	chr9	3006765	3007176	Mir101c
Hx versus Nx	999	chr9	3000393	3001390	Mir101c
Hx versus Nx	306	chrJH584304.1	90204	90508	
Hx versus Nx	443	chrJH584304.1	87730	88171	

Supp. Table 2. Enriched genomic binding sites for Sirt2.

Complete list of identified genomic binding sites (referred to as peaks) for Sirt2 in dissected WM tissue from Nx and Hx-treated postnatal WT mouse brains. Three comparisons were performed: Nx Sirt2 ChIP versus Nx IgG negative control samples (Nx versus IgG), Hx Sirt2 ChIP versus Hx IgG negative control samples (Hx versus IgG), and Hx Sirt2 ChIP versus Nx Sirt2 ChIP (Hx versus Nx).
