# Supplementary material for: Sirt2 promotes white matter oligodendrogenesis during development and in models of neonatal hypoxia
Source: Nat Commun. 2022 Aug 15;13:4771. doi: 10.1038/s41467-022-32462-2 (PMC9378658; doi:10.1038/s41467-022-32462-2)
Supplement: Supplementary file 2 — Reporting Summary [file 41467_2022_32462_MOESM2_ESM.pdf]

nature portfolio

Corresponding author(s): Vittorio Gallo

Last updated by author(s): Jul 19, 2022

Reporting Summary

Nature Portfolio wishes to improve the reproducibility of the work that we publish. This form provides structure for consistency and transparency in reporting. For further information on Nature Portfolio policies, see our Editorial Policies and the Editorial Policy Checklist.

Statistics

For all statistical analyses, confirm that the following items are present in the figure legend, table legend, main text, or Methods section.

n/a	Confirmed	
☐	☒	The exact sample size (*n*) for each experimental group/condition, given as a discrete number and unit of measurement
☐	☒	A statement on whether measurements were taken from distinct samples or whether the same sample was measured repeatedly
☐	☒	The statistical test(s) used AND whether they are one- or two-sided<br>*Only common tests should be described solely by name; describe more complex techniques in the Methods section.*
☒	☐	A description of all covariates tested
☐	☒	A description of any assumptions or corrections, such as tests of normality and adjustment for multiple comparisons
☐	☒	A full description of the statistical parameters including central tendency (e.g. means) or other basic estimates (e.g. regression coefficient) AND variation (e.g. standard deviation) or associated estimates of uncertainty (e.g. confidence intervals)
☐	☒	For null hypothesis testing, the test statistic (e.g. *F*, *t*, *r*) with confidence intervals, effect sizes, degrees of freedom and *P* value noted<br>*Give P values as exact values whenever suitable.*
☒	☐	For Bayesian analysis, information on the choice of priors and Markov chain Monte Carlo settings
☒	☐	For hierarchical and complex designs, identification of the appropriate level for tests and full reporting of outcomes
☒	☐	Estimates of effect sizes (e.g. Cohen's *d*, Pearson's *r*), indicating how they were calculated

Our web collection on statistics for biologists contains articles on many of the points above.

Software and code

Policy information about availability of computer code

Data collection	Immunohistochemical data was collected using Leica LASX, Zeiss LSM or Zen 2.3 or software. Brightfield images of human H&E stains were collected using CellSense 4.1 software. ChIP-sequencing was performed on Novaseq 6000 (Illumina).
Data analysis	Images were viewed and analzyed using FIJI ImageJ softare (version 2.0.0, NIH). Data was analyzed using Microsoft Excel (version 16.16.4) and Graphpad/Prism (version 9). ChIPseq data was analyzed using bowtie (v2.4.4), Samtools (v1.7), Picard (v2.26.10), Macs2 (v2.2.7.1), and Homer (v4.11). Figures were prepared using Adobe Photoshop (Adobe Creative Suite C56 Design Standard) and CorelDraw 2018 (Version 20.1.0.708).

For manuscripts utilizing custom algorithms or software that are central to the research but not yet described in published literature, software must be made available to editors and reviewers. We strongly encourage code deposition in a community repository (e.g. GitHub). See the Nature Portfolio guidelines for submitting code & software for further information.

Data

Policy information about availability of data

All manuscripts must include a data availability statement. This statement should provide the following information, where applicable:

- Accession codes, unique identifiers, or web links for publicly available datasets
- A description of any restrictions on data availability
- For clinical datasets or third party data, please ensure that the statement adheres to our policy

ChIP-seq data generated from this study have been deposited in the NCBI Sequence Read Archive (SRA) database with the accession code PRJNA773120 (https://www.ncbi.nlm.nih.gov/bioproject/PRJNA773120). ChIP-seq reads were aligned to the mouse reference genome (Genome Reference Consortium Mouse Build-mm10). Source data for Figures 1-8 and Supplementary Figures 1-10 are provided with the paper.

Field-specific reporting

Please select the one below that is the best fit for your research. If you are not sure, read the appropriate sections before making your selection.

- [x] Life sciences
- [ ] Behavioural & social sciences
- [ ] Ecological, evolutionary & environmental sciences

For a reference copy of the document with all sections, see nature.com/documents/nr-reporting-summary-flat.pdf

Life sciences study design

All studies must disclose on these points even when the disclosure is negative.

Sample size	The sample sizes for these experiments were based on similar experiments done in the lab (Jablonska et al. 2016). The sample size of all experiments in this study was a minimum of 3 animals, as is standard in the field.
Data exclusions	No data were excluded.
Replication	All experiments were replicated on numerous animals, collected across many days. The primary cellular and molecular findings were successfully replicated 3-4 times in both in vivo mouse studies and in vitro cell culture assays.
Randomization	Primary white matter cells were randomly transfected with either control siRNA, Sirt2 siRNA, or Sirt1 siRNA. For in vivo studies: allocation into experimental groups was primarily dependent on genotype (mouse experiments) or age at birth (human tissue analysis). Within the different mouse strains, litters were randomly assigned to normoxic or hypoxic experimental conditions. All mice (both control and experimental groups) received tamoxifen injections for in vivo studies.
Blinding	No blinding was performed for in vivo mouse studies because of the need to obtain sufficient biological replicates of specific genotypes for each experimental condition. Human tissue was stained and imaged blind. Analysis of ChIP-seq data required unblinding to perform the correct comparisons.

Reporting for specific materials, systems and methods

We require information from authors about some types of materials, experimental systems and methods used in many studies. Here, indicate whether each material, system or method listed is relevant to your study. If you are not sure if a list item applies to your research, read the appropriate section before selecting a response.

Materials & experimental systems

n/a	Involved in the study	
[ ]	[x]	Antibodies
[x]	[ ]	Eukaryotic cell lines
[x]	[ ]	Palaeontology and archaeology
[ ]	[x]	Animals and other organisms
[ ]	[x]	Human research participants
[x]	[ ]	Clinical data
[x]	[ ]	Dual use research of concern

Methods

n/a	Involved in the study	
[ ]	[x]	ChIP-seq
[x]	[ ]	Flow cytometry
[x]	[ ]	MRI-based neuroimaging

Antibodies

Antibodies used	The following antibodies were used for mouse tissue staining: NG2 (1:250, Chemicon, AB5320), GFAP (1:500, Chemicon, MAB3402), Olig2 (1:200, Abcam, ab33437), CC1 (1:500, CalBiochem, OP80), Sirt2 (1:200, ABCAM, ab67299), Iba1 (1:200, WAKO, 019-19741), PDGFRα (1:250, BD Bioscience 558774), CNP (1:100, Biolegend, 836404), MBP (1:200, Biolegend, 808402). Appropriate secondary antibodies were used as follow: TRITC-conjugated AffiniPure Goat Anti-Mouse IgG (H+L), FITC-conjugated AffiniPure Goat Anti-Rabbit IgG, TRITC-conjugated AffiniPure Goat Anti-Mouse IgM (all 1:200, 115-025-146, 111-095-008, 115-025-020, respectively, all from Jackson ImmunoResearch).<br>The following antibodies were used for mouse cell culture staining: GalC (1:200, Galactocerebroside; Abcam ab142) and Olig2 (1:200, Abcam, ab9610).<br>The following antibodies were used for human tissue staining: Olig2 (1:200, R&D #AF2418), Sirt2 (1:200, Abcam #211033), Biotin-SP (1:500, Jackson, 705-065-147), and streptavidin-HRP (1:2000, Perkin Elmer, NEL750).<br>The following antibodies were used for Western blots: anti-Cdk4 (1:5000,Santa Cruz, sc-260), -cyclin D (1:5000, Santa Cruz, sc25765), -p107 (1:2000, Santa Cruz, sc-65221), -E2F4 (1:2000, Santa Cruz, sc-866), -p27Kip1 (1:1000, Santa Cruz, sc-528), -Sirt1 (1:1000, Santa Cruz, sc-15404), -acetyl-lysine (1:1000, Cell Signaling, 9681), -FoxO1 (1:5000, Cell Signaling, L27), -Actin (1:10000, Chemicon, MAB 1501R), -Sirt2 (1:5000, Abcam, ab211033), -Sirt2 phospho Ser331 (1:5000, Active Motif, 61363), -p35 (1:5000, Abcam, ab123048), -Cdk5 (1:5000, Invitrogen, PAS-28795), and -p21Cip1 (1:1000, CalBiochem, OP76). Bands were detected with appropriate horseradish peroxide (HRP)-conjugated secondary antibodies and reacted with chemiluminescent ECL substrate (Amersham, RPN2132).
Validation	All primary antibodies have validated for immunohistochemistry or Western blot for use on mouse or human. More information,

including citations, can be found on manufactures website. The Sirt2 antibody used for ChIP-seq was validated by Western blot and immunostaining of cultured cells (Supplemental figure 9).

Animals and other organisms

Policy information about studies involving animals; ARRIVE guidelines recommended for reporting animal research

Laboratory animals	Species: mouse (Mus musculus). Strains from Jackson: WT (C57BL/6), Sirt2STOP, Sirt1fl/fl, Sirt2fl/fl, PDGFRCreER, PLPCreER (Jackson Laboratory cat #003548, 029604, 029603, 030835, 032770, 005975), CD1 (Charles River Crl:CD1(ICR)). CNP-EGFP mice were previously generated in our lab. Sirt2 floxed mice were also obtained from Dr. Nave at Max Planck Institute in Germany.<br>Sex: both males and females were used. Age: Mice were sacrificed at P11-P18 for analysis, and the exact time point is written in the manuscript text and figure legends. Both male and female mice were analyzed for all experiments. Mice were maintained in animal facility under a 12h dark–light cycle, and constant temperature (20–26°C) and humidity maintenance (40–60%).
Wild animals	The study did not involve wild animals.
Field-collected samples	The study did not involve samples collected from the field.
Ethics oversight	All animal procedures were approved by the Institutional Animal Care and Use Committee (IACUC) of the Children's National Health System (protocol #30473).

Note that full information on the approval of the study protocol must also be provided in the manuscript.

Human research participants

Policy information about studies involving human research participants

Population characteristics	A total of 8 human subjects were enrolled from NIH Neurobiobank and Children's National Pathology Department. Of these 8 autopsy cases of infants – 4 had a diagnosis of preterm birth less than 32 weeks of gestation and 4 infants were born at term (>37 weeks of gestation). Sex, age at birth, adjusted age, and cause of death are reported are Supplementary Table 1.
Recruitment	Post-mortem human tissue was selected based on the age at birth.
Ethics oversight	The experiments with human tissue were approved by the Institutional Review Board at Children's National Hospital (IRB#00011850).

Note that full information on the approval of the study protocol must also be provided in the manuscript.

ChIP-seq

Data deposition

- [x] Confirm that both raw and final processed data have been deposited in a public database such as GEO.
- [x] Confirm that you have deposited or provided access to graph files (e.g. BED files) for the called peaks.

Data access links<br>*May remain private before publication.*	https://www.ncbi.nlm.nih.gov/bioproject/PRJNA773120
Files in database submission	ChIP-seq of Normoxic white matter with IgG antibody (SRX12709563), ChIP-seq of Hypoxic white matter with IgG antibody (SRX12709564), ChIP-seq of Normoxic white matter with Sirt2 antibody (SRX12709561), ChIP-seq of Hypoxic white matter with Sirt2 antibody (SRX12709562).
Genome browser session<br>(e.g. UCSC)	No longer applicable.

Methodology

Replicates	Two samples (Sirt2 antibody treated vs IgG) were sequenced for each of hypoxic and normoxic conditions.
Sequencing depth	About 107.8 million total paired-end (PE75) reads were generated, averaging about 27 million reads per sample. Overall, 52-90% reads were aligned in each of the four samples, of which 39-40% were uniquely mapped.
Antibodies	anti-Sirt2 antibody (Santa Cruz, sc211033) or IgG negative control antibody.
Peak calling parameters	Macs2 (2.2.7.1) was for peak calling with default parameters.
Data quality	The ENCODE ChipSeq pipeline was followed. Briefly, Bowtie2 (v2.4.4) was used for alignment with "very-sensitive" preset parameters to mouse genome reference (mm10). Then, aligned reads were filtered with Samtools (v1.7), keeping ones with quality score (Q-score) at least 30. Blacklisted regions of the mouse genome were removed with Bedtools (v2.30.0). Duplicated reads were marked with Piccard (v2.26.10) MarkDuplicate function and removed. 19 peaks were identified at fold change greater than 5 and FDR 10%, comparing hypoxic and normoxic samples treated with antibody.
Software	Bowtie2 (v2.4.4) was used for alignment. Homer (v4.11) was used for peak annotation.
